# Uncovering the unexplored diversity of thioamidated ribosomal peptides in Actinobacteria using the RiPPER genome mining tool

**DOI:** 10.1093/nar/gkz192

**Published:** 2019-03-27

**Authors:** Javier Santos-Aberturas, Govind Chandra, Luca Frattaruolo, Rodney Lacret, Thu H Pham, Natalia M Vior, Tom H Eyles, Andrew W Truman

**Affiliations:** Department of Molecular Microbiology, John Innes Centre, Norwich, Norfolk NR4 7UH, UK

## Abstract

The rational discovery of new specialized metabolites by genome mining represents a very promising strategy in the quest for new bioactive molecules. Ribosomally synthesized and post-translationally modified peptides (RiPPs) are a major class of natural product that derive from genetically encoded precursor peptides. However, RiPP gene clusters are particularly refractory to reliable bioinformatic predictions due to the absence of a common biosynthetic feature across all pathways. Here, we describe RiPPER, a new tool for the family-independent identification of RiPP precursor peptides and apply this methodology to search for novel thioamidated RiPPs in Actinobacteria. Until now, thioamidation was believed to be a rare post-translational modification, which is catalyzed by a pair of proteins (YcaO and TfuA) in Archaea. In Actinobacteria, the thioviridamide-like molecules are a family of cytotoxic RiPPs that feature multiple thioamides, which are proposed to be introduced by YcaO-TfuA proteins. Using RiPPER, we show that previously undescribed RiPP gene clusters encoding YcaO and TfuA proteins are widespread in Actinobacteria and encode a highly diverse landscape of precursor peptides that are predicted to make thioamidated RiPPs. To illustrate this strategy, we describe the first rational discovery of a new structural class of thioamidated natural products, the thiovarsolins from *Streptomyces varsoviensis*.

## INTRODUCTION

Microorganisms have provided humankind with a vast plethora of specialized metabolites with invaluable applications in medicine and agriculture (1). The advent of widespread genome sequencing has shown that the metabolic potential of bacteria had been substantially underestimated, as their genomes contain many more biosynthetic gene clusters (BGCs) than known compounds (2,3). Much of this enormous potential is either unexplored or undetectable under laboratory culture conditions, and is likely to include structurally novel bioactive specialized metabolites. Among the main classes of specialized metabolites produced by microorganisms, the ribosomally synthesized and post-translationally modified peptides (RiPPs) (4) may harbor the largest amount of unexplored structural diversity. This is due to the inherent difficulties related to the *in silico* prediction of their BGCs, as RiPP biosynthetic pathways lack any kind of universally shared feature apart from the existence of a pathway-specific precursor peptide.

RiPP BGCs can be identified by the co-occurrence of specific RiPP tailoring enzymes (RTEs) alongside a precursor peptide that contains sequence motifs that are characteristic of a given RiPP family. This makes it relatively simple to identify further examples of known RiPP families (5,6), but the identification of currently undiscovered RiPP families remains a significant unsolved problem. Unlike specialized metabolites such as polyketides, non-ribosomal peptides and terpenes, there are no genetic features that are common to all RiPP BGCs to aid in their identification. Furthermore, genes encoding precursor peptides are often missed during genome annotation due to their small size, yet the reliable prediction of precursor peptides constitutes a crucial task, as this starting scaffold is essential for RiPP structural prediction. Numerous analyses of specific RiPP classes signal the existence of a wide array of uncharacterized RiPP families (7–9), but currently available prediction tools still rely on precursor peptide features or generic RTEs that are associated with known RiPP families (10–14).

YcaO domain proteins are a widespread superfamily of enzymes with an intriguing catalytic potential in RiPP biosynthesis (15). These were originally shown to be responsible for the introduction of oxazoline and thiazoline heterocycles in the precursor peptide backbone of microcins (16), and were very recently demonstrated to catalyze the formation of the macroamidine ring of bottromycin (17–19). YcaO proteins act as cyclodehydratases, activating the amide bond substrate by nucleophilic attack, which is followed by ATP-driven O-phosphorylation of the hemiorthoamide intermediate and subsequent elimination of phosphate. In most azoline-containing RiPPs, this catalytic activity requires a partner protein (E1-like or Ocin-ThiF-like proteins that are clustered with or fused to the YcaO domain), which acts as a docking element to bring the precursor peptide to the active site of the cyclodehydratase (15). YcaO proteins can also act as standalone proteins, as in bottromycin biosynthesis (18,19), and many YcaO proteins are encoded in genomes without E1-like or Ocin-ThiF-like partner proteins (9,15), including in the BGCs of thioviridamide-like molecules (6,20–24).

**Figure 1.**
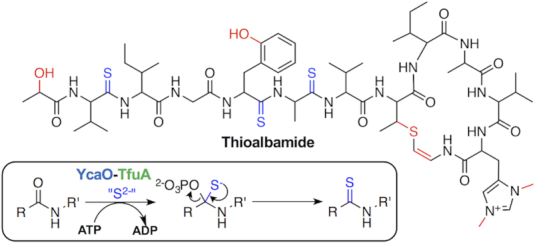
An example of a thioviridamide-like molecule, thioalbamide, and inset, a proposed biochemical route to thioamides. Thioamides are highlighted in blue and other post-translational modifications are colored red.

Thioviridamide and related compounds are cytotoxic RiPPs that contain multiple thioamide groups (Figure 1), but no azole or macroamidine rings. Thioamides are rare in nature (25–31) and it has been hypothesized that YcaO proteins could be responsible for this rare amide bond modification in thioviridamide biosynthesis, potentially in cooperation with TfuA domain proteins (15) (Figure 1). This protein pair has been identified elsewhere in nature, including in archaea, where they are involved in the ATP-dependent thioamidation of a glycine residue of methyl-coenzyme M reductase (32,33). We therefore hypothesized that the identification of *tfuA*-like genes could be employed as a rational criterion for the identification of BGCs responsible for the production of novel thioamidated RiPPs in bacteria.

An exploration of the diversity of *tfuA*-containing BGCs required methodology to identify precursor peptides that have no homology to known precursor peptides. Here, we report RiPPER (RiPP Precursor Peptide Enhanced Recognition), a method for the identification of precursor peptides that requires no information about RiPP structural class (available at https://github.com/streptomyces/ripper). This evaluates regions surrounding any putative RTE for short open reading frames (ORFs) based on the likelihood that these are truly peptide-coding genes. Peptide similarity networking is then used to identify putative RiPP families. We apply this methodology to identify RiPP BGCs encoding TfuA proteins in Actinobacteria, which reveals a highly diverse landscape of BGC families that are predicted to make thioamidated RiPPs. This analysis informed the discovery of the thioamidated thiovarsolins from *Streptomyces varsoviensis*, which are predicted to belong to a wider family of related thioamidated RiPPs and represents the first rational discovery of a new family of thioamidated compounds from nature.

## MATERIALS AND METHODS

### Chemicals

Unless otherwise specified, chemicals were purchased from Sigma-Aldrich, and enzymes from New England Biolabs. Molecular biology kits were purchased from Promega and GE Healthcare.

### Strains and culture conditions

*Streptomyces varsoviensis* DSM 40346 was acquired from the German Collection of Microorganisms and Cell Cultures (DSMZ, Germany) and used as genetic source for the thiovarsolin gene cluster. *Streptomyces coelicolor* M1146, *S. coelicolor* M1152 (34) and *Streptomyces lividans* TK21 were used as heterologous expression hosts. All culture media and primers used in this work are described in full in the Supplementary Methods. Unless otherwise specified, all *Streptom*yces strain were grown in SFM (solid) and TSB (liquid) media at 28°C. Spores and mycelium stocks were kept at −20°C and −80°C in 20% glycerol. *Saccharomyces cerevisiae* VL6–48N (35) was used for transformation-associated recombination (TAR) cloning and was grown at 30°C with shaking at 250 rpm in YPDA medium. Recombinant yeast selection was performed using selective media SD+CSM-Trp complemented with 5-fluoorotic acid (Fluorochem, 1 mg ml^−1^). Yeast cell stocks were kept at −80°C in 20% glycerol. *Escherichia coli* DH5α was used for standard DNA manipulations. *E. coli* DH5α BT340 was used for Flp-*FRT* recombination. *E. coli* BW25113/pIJ790 was used for Lambda-Red mediated recombination. *E. coli* ET12567/pR9604 and *E. coli* ET12567/pUZ8002 were used to transfer DNA to *Streptomyces* by intergeneric conjugation. All *E. coli* strains were grown in LB medium at 37°C unless specified by particular protocols (pIJ790-carrying strains were grown at 30°C for plasmid replication, and Flp-*FRT* recombination was performed at 42°C). *Escherichia coli* hygromycin selection was performed in DNAm (solid) and DNB (liquid) media. *E. coli* cell stocks were kept at −20°C and −80°C in 20% glycerol.

### RiPPER details

RiPPER consists of a series of Perl scripts that require the RODEO2 Python script (13,14), BioPerl (36), a locally installed Pfam database (37,38) and a modified build of Prodigal (39) (which we name Prodigal-short) to operate. Analysis parameters for RiPPER are defined in an associated configuration file (local.conf), which can be modified to optimize the genome mining process. EGN (Evolutionary Gene and genome Network) (40) was used to construct protein similarity networks, which were visualized using Cytoscape 2.8.3 (41). Further information is provided in the documentation provided with the RiPPER scripts at https://github.com/streptomyces/ripper. For ease of use, a Docker container is provided that contains all features required for using RiPPER. This is available at https://hub.docker.com/r/streptomyces/ripdock/ along with instructions on installation and usage. A workflow for using RiPPER is described below.

### Workflow for RiPPER

Below is a summary of the RiPPER workflow, which has been developed for gene cluster visualization in Artemis (42) (Supplementary Figure S1). Where relevant, default analysis parameters are listed. These are all customizable from the local.conf configuration file associated with a given RiPPER analysis.

Using RODEO (13,14), accession numbers for a set of putative RiPP tailoring enzymes (RTEs) are used to obtain nucleotide regions (as GenBank files) centered on the tailoring enzyme, which is highlighted as a green gene for clarity in Artemis. 25 kb regions were obtained for the TfuA analysis (flankLen = 12.5 kb), and 35 kb regions were obtained for the known RiPP families (flankLen = 17.5 kb, default).

1. Every retrieved genomic region is subjected to RODEO analysis to obtain a RODEO output for each input accession, as well as Pfam domain data across the gene cluster.
2. GenBank files are then analyzed using a specially built version of Prodigal (39), which we call Prodigal-short. This is configured to find genes as short as 60 nucleotides instead of the usual size cut-off of 90 nucleotides.
3. For all the genes found by Prodigal-short the following is done:
  - The Prodigal score is enhanced if the gene is on the same strand as the tailoring enzyme (sameStrandReward, default = 5).
  - Genes are only retained for analysis if they overlap with existing annotated genes by 20 nucleotides or less.
  - RiPPER uses Prodigal-short to only identify putative ORFs within a likely size window for precursor peptide genes. Therefore, genes are only retained for analysis if the length of the encoded peptide is between minPPlen and maxPPlen. A window of 20–120 AA (default) was used in all analyses in this study.
  - If a gene is not filtered out in the above steps, it is annotated in the GenBank file and its distance from the tailoring enzyme is determined.
  - All putative genes identified are provided in the resulting GenBank file and are color-coded from pale red (low score) to bright pink (high score) (Supplementary Figure S1). Scoring criteria are viewable in Artemis as notes for each putative gene.
  - RiPPER also retrieves and scores genes that were already annotated if they encode peptides below the maxPPlen (default = 120 AA). This means that annotated precursor peptides are also retrieved for downstream analysis.
4. The resulting annotated GenBank files can be viewed in Artemis at this stage for manual identification of RiPP precursor peptides.
5. If the gene is within a specified distance (maxDistFromTE) from the RTE, it is included in the output list and also saved in a Sqlite3 table. A distance of ±8 kb is used as default.
6. Within this region, the top scoring short peptides (no lower score threshold) are retrieved. The number retrieved is defined by fastaOutputLimit (default = 3) In addition, any further peptides with Prodigal scores over a threshold (prodigalScoreThresh) within this region are retrieved. A score threshold of 15 was used in the TfuA analysis and a score threshold of 7.5 (default) was used in the analysis of known RiPP families.
7. All retrieved peptides are analyzed for Pfam domains, and all information is tabulated alongside various associated data (tailoring enzyme accession, strain, peptide sequence, distance from tailoring enzyme, coding strand in relation to tailoring gene, Prodigal score) in a tab-separated out.txt file. All data are collated in a single file if multiple genomic regions are analyzed in parallel.
8. All peptides identified by RiPPER across the entire Genbank file that were not retrieved in step 6 (no distance or score threshold) are searched for characterized precursor peptide domains (38). Data for these peptides is then tabulated in a tab-separated distant.txt file.
9. Optional follow-on analysis: protein similarity networking and BGC comparative analysis. Protein similarity networking does not form part of the automated RiPPER workflow, but this does assist with the identification of authentic precursor peptides. The RiPPER output includes fasta files (out.faa and distant.faa) for all retrieved peptides that are compatible for analysis with EGN (40). The following settings were used for all analyses: *E*-value threshold = 10, hit identity threshold = 40%, hit covers at least 35% of the shortest sequence, minimum hit length = 15 AA. The resulting networks were visualized using Cytoscape 2.8.3 (41), where data obtained from RiPPER were imported as node attributes. The similarity between BGCs associated with the same network was assessed using MultiGeneBlast (43). Peptides from each network were aligned using MUSCLE (44) and alignments were visualized using ESPript 3.0 (45).

### Identification of precursors to lasso peptides, microviridins and thiopeptides

Studies by Tietz *et al.* (13), Ahmed *et al.* (46) and Schwalen *et al.* (14) had previously used RTE accessions to mine for precursors to lasso peptides, microviridins and thiopeptides, respectively. The same accession codes were used to mine for precursor peptides using RiPPER (Supplementary Datasets 1–3), although not all accessions could be retrieved as some records no longer exist on NCBI. RiPPER was run using analysis parameters as described above and the results are described in Table 1. Peptide similarity networking was carried out using EGN (as described above), which provided large networks for each dataset (Network 1, Supplementary Figures S2–S4, Supplementary Datasets 1–3). To determine the ability of RiPPER to retrieve authentic precursor peptide sequences, a bespoke script was used to compare the RiPPER outputs with the prior studies.

**Table 1.** Comparison of RiPPER with prior studies on the identification of RiPP precursor peptides

		Generic RiPPER search		RiPPER including HMM search		Network 1 data from RiPPER analysis		
RiPP class^a^	No. of RTEs used in RiPPER search	Total peptides retrieved	Match with prior data^b^	Total peptides retrieved	Match with prior data^b^	Total peptides in network	Match with prior data^b^	Additional HMM hits
Lasso peptides	1198	4503	1056/1122 (94.1%)	4558	1063/1122 (94.7%)	1211^c^	934/1122 (83.2%)	125
Microviridins	159	586	270/280 (96.4%)	596	270/280 (96.4%)	270	269/280 (96.1%)	1
Thiopeptides	486	1526	438/591 (74.1%)	1675	549/591 (92.9%)	690	543/591 (91.9%)	75

^a^Data obtained for lasso peptides from ref. 13, microviridins from ref. 46 and thiopeptides from ref. 14.

^b^These numbers are sometimes greater than the number of RTEs used in the RiPPER search due to the identification of multiple precursor peptides per BGC.

^c^Proteins with PqqD domains removed.

### TfuA-like protein retrieval and phylogenetic analysis

The NCBI Conserved Domain Architecture Retrieval Tool (CDART) (47) was used to retrieve all TfuA domain protein sequences from the phylum Actinobacteria in the NCBI non-redundant protein sequence database. These 325 proteins were manually assessed by Pfam analysis for TfuA domains, which resulted in the removal of five proteins from this dataset. To limit the overrepresentation of highly similar proteins in an analysis of phylogeny and gene cluster diversity, ElimDupes (https://www.hiv.lanl.gov/content/sequence/elimdupesv2/elimdupes.html) was used to remove proteins with at least 99% identity to each other from the dataset to leave one representative protein. This provided a dataset of 229 TfuA domain proteins. Three proteins that contained fused YcaO and TfuA domains were removed for phylogenetic analysis, along with one (KZS83678.1) that is truncated. The standalone TfuA domain protein dataset (225 proteins) was aligned using MUSCLE 3.8.31 (44) with default settings. The resulting alignment was used to construct a maximum likelihood tree using RAxML-HPC2 on XSEDE (with 100 bootstrap replications) on the CIPRES Science Gateway (https://www.phylo.org/). The tree was visualized using the interactive Tree Of Life (iTOL) (48) (Supplementary Dataset 5). The statistical analysis of the lengths of predicted precursor peptides is described in the Supplementary Methods.

### TAR cloning and heterologous expression of the thiovarsolin gene cluster

A vector to capture the thiovarsolin gene cluster from *S. varsoviensis* genomic DNA (gDNA) was constructed using yeast assembly between a linearized pCAP03 vector (49) and two single-strand oligonucleotides (TARvar-1 and TARvar-2). Oligonucleotides had 35 nucleotide homology sequences with pCAP03 and were designed to generate a vector with 50 nucleotide homology sequences with upstream and downstream regions of the gene cluster either side of a PmeI restriction site. pCAP03 was digested with XhoI and NdeI, and the linearized plasmid and ss-oligos (1:10 ratio) were transformed into *S. cerevisiae* VL6–48N by lithium acetate/polyethylene glycol 3350 mediated transformation. For yeast-colony PCR, each colony was resuspended in 50 μl 1 M sorbitol (Fisher) and 2 μl of zymolyase (5 U μl^−1^) added to each cell suspension and incubated at 30°C for 1 hour. Cell suspensions were then boiled for 10 min, centrifuged (15 s, 1000 × *g*) and 1 μl of the supernatant was analyzed by PCR.

To transfer the plasmids from yeast into *E. coli*, colonies of yeast were grown in 10 ml of liquid SD+CSM-Trp for 18 h at 250 rpm, 30°C. Cells were harvested by centrifugation (5 min, 1789 × *g*), and resuspended in 200 μl 1 M sorbitol plus 2 μl of zymolyase (5 U μl^−1^). Cell suspensions were incubated at 30°C for 1 hour to produce spheroplasts, which were then pelleted (10 min, 600 × *g*). The supernatant was aspirated, and plasmid DNA extracted from the pellet using a standard Wizard miniprep protocol (Promega). 1 μl plasmid DNA was then transformed into *E. coli* DH5α by electroporation and selected with kanamycin (50 μg ml^−1^) Colonies containing the correct capture vector were identified by PCR (primers: CAP03_check-fw and CAP03_check-rv), and the plasmid was isolated and confirmed by sequencing.

gDNA from *S. varsoviensis* was digested with EcoRV and ScaI, and the pCAP03-derived capture vector was linearized between the capture arms with PmeI. These were both then introduced into *S. cerevisiae* VL6–48N by spheroplast polyethylene glycol 8000 transformation. Successful gene cluster capture by pCAP03 was confirmed by colony PCR (primers: TARcheck-fw and TARcheck-rv). The plasmids from three positive clones were recovered and transformed into electrocompetent *E. coli* DH5α for amplification and further restriction analysis of the purified construct (pTARvar). *E. coli* ET12567/pR9604 was transformed with pTARvar by electroporation, and transformants were then used to transfer pTARvar into *S. coelicolor* (M1146 and M1152) and *S. lividans* TK21 by intergeneric conjugation. Nalidixic acid (25 μg ml^−1^) and kanamycin-resistant (50 μg ml^−1^) exconjugants containing integrated pTARvar (*S. coelicolor* M1146-TARvar, *S. coelicolor* M1152-TARvar and *S. lividans* TK21-TARvar) were verified by PCR using GoTaq polymerase (Promega) (primers: TAR_check-fw and TAR_check-rv).

### Fermentation conditions for metabolite screening

Seed cultures of *S. coelicolor* M1146-TARvar, *S. coelicolor* M1152-TARvar and *S. lividans* TK21-TARvar were obtained by fermentation in a 250 ml flask containing 50 mL of TSB for 72 h. 250 μl seed culture was used to inoculate 5 ml of a variety of culture media (TSB, BPM, GYM, MI, TPM, E25; see Supplementary Methods) in 50 ml conical centrifuge tubes with caps replaced by foam bungs. Control strains carrying a genome-integrated empty pCAP03 vector were cultured in the same way for comparison. All fermentations were conducted in triplicate and incubated at 28°C with shaking at 250 rpm. Culture samples (500 μl) were taken at 72 and 168 h, mixed with one volume of methanol and agitated for 30 min at room temperature. These mixtures were then centrifuged (15,871 × *g*, 30 min) and 600 μl of the resulting supernatant was transferred to glass vials for liquid chromatography–mass spectrometry (LC–MS) analysis. Details on the large-scale fermentation, isolation and structural elucidation of thiovarsolins A and B are described in the Supplementary Methods.

### LC–MS analysis

Spectra were obtained using a Shimadzu Nexera X2 UHPLC coupled to a Shimadzu IT-TOF mass spectrometer. Samples (5 μl) were injected onto a Phenomenex Kinetex 2.6 μm XB-C18 column (50 mm × 2.1 mm, 100 Å) set at a temperature of 40°C and eluting with a linear gradient of 5–95% acetonitrile in water + 0.1% formic acid over 6 minutes with a flow-rate of 0.6 ml min^−1^. Positive mode mass spectrometry data was collected between *m/z* 200 and 2000, and MS^2^ data was collected using collision-induced dissociation of the most abundant singly charged species in a scan, with an exclusion time of 0.8 seconds. Untargeted comparative metabolomics was carried out on triplicate data using Profiling Solution 1.1 (Shimadzu) with an ion *m/z* tolerance of 100 mDa, a retention time tolerance of 0.1 min and an ion intensity threshold of 100,000 units.

For the accurate mass measurement of the thiovarsolins, high-resolution mass spectra were acquired by LC–MS on a Synapt G2-Si mass spectrometer equipped with an Acquity UPLC (Waters). Samples were injected onto an Acquity UPLC^®^ BEH C18 column, 1.7 μm, 1 × 100 mm (Waters) and eluted with a gradient of (B) acetonitrile/0.1% formic acid in (A) water/0.1% formic acid with a flow rate of 0.08 ml min^−1^ at 45°C. The concentration of B was kept at 1% for 2 min followed by a gradient up to 30% B in 4 min. MS data were collected with the following parameters: resolution mode, positive ion mode, scan time 0.5 s, mass range *m/z* 50–1200 (calibrated with sodium formate), capillary voltage = 3.0 kV; cone voltage = 40 V; source temperature = 120°C; desolvation temperature = 350°C. Leu-enkephalin peptide was used to generate a lock-mass calibration with *m/z* = 556.2766 measured every 30 s during the run.

### Deletion of genes in the thiovarsolin biosynthetic gene cluster

The mutational analysis of the thiovarsolin BGC was performed using an *E. coli*-based Lambda-Red-mediated PCR-targeting strategy (50), which allowed the substitution of genes or groups of genes in pTARvar by a PCR-generated cassette containing the apramycin resistance gene *aac(3)IV*. Given the presence of an *oriT* in the original pCAP03 vector, the upstream primer design was modified with respect to the original protocol in order to exclude a second *oriT* from the PCR-targeting resistance cassette and avoid undesired recombinations. Therefore, resistance cassettes were PCR amplified using pIJ773 as template (see primers in Supplementary Table S3 and mutants in Supplementary Table S4). In the case or *varA*, an additional in-frame deletion mutant affecting only the core precursor peptide was created employing a pIJ773-derived cassette lacking OriT (pIJ773 Δ*oriT*) but preserving both *FRT* recombination sites (primers RD1 and RD3), which allowed the elimination of the apramycin resistance cassette after Flp-*FRT* recombination in *E. coli* DH5α BT340 and the creation of a clean *varA* mutant (Δ*varA*_clean). The PCR-targeting mutant versions of pTARvar were transferred to *S. coelicolor* M1146 by *E. coli* ET12567/pUZ8002-mediated intergeneric conjugation and selected by resistance to nalidixic acid (25 μg ml^−1^), kanamycin (50 μg ml^−1^) and, when required, apramycin (50 μg ml^−1^).

Constructs for the complementation of mutants showing differences in thiovarsolin production in comparison to *S. coelicolor* M1146-TARvar (Δ*varA*, Δ*varY*, Δ*varT* and Δ*varO*) were obtained by high-fidelity PCR amplification (Herculase II, Agilent) of each of these genes (primers CP1 and CP2 for *varA*, CP3 and CP4 for *varA*p, CP5 and CP6 for *varY*, CP7 and CP8 for *varT*, and CP9 and CP10 for *varO*), digestion of the PCR product with NdeI and HindIII and cloning by ligation (T4 DNA ligase, Invitrogen) into NdeI – HindIII digested pIJ10257 (51). Ligation mixtures were transformed into chemically competent *E. coli* DH5α, plasmids were recovered by alkaline lysis and then sequenced. The resulting plasmids (pJ10257-*varA*, pIJ1027-*varA*p, pIJ10257-*varY*, pIJ10257-*varT* and pIJ10257-*varO*) were introduced into the corresponding *S. coelicolor* M1146-var mutants by *E. coli* ET12567/pUZ8002-mediated intergeneric conjugation. Exconjugants were selected by resistance to nalidixic acid (25 μg ml^−1^), kanamycin (50 μg ml^−1^), hygromycin (50 μg ml^−1^) and, when required, apramycin (50 μg ml^−1^). The construction of a minimal thiovarsolin gene cluster (pIJ10257-*varA*p*YT*) and the site-directed mutagenesis of *varA* are described in the Supplementary Methods.

## RESULTS AND DISCUSSION

### Development of a family-independent RiPP genome mining tool

Within a given RiPP family, all BGCs usually encode at least one tailoring enzyme and one precursor peptide that each feature domains conserved across the RiPP family (4). This has led to the development of genome mining methodology that can identify these well-characterized RiPP families with high accuracy (10–13). However, there is a growing number of widespread RiPP BGCs with little or no homology to known RiPP BGCs (7,52). Theoretically, backbone modifications such as thioamidation or epimerization (53) can occur on any residue. In addition, well-characterized RiPP tailoring enzymes can be associated with unusual precursor peptides that lack homology to known RiPP classes (9). We therefore sought to develop a method to identify likely precursor peptides that was independent of precursor peptide sequence and could be applicable for any RiPP family. The starting point for this method was to employ the functionality of RODEO (13,14) to identify genomic regions associated with a series of putative RTEs. RODEO uses a mixture of heuristic scoring and support vector machine classification to identify precursor peptides for lasso peptides (13) and thiopeptides (14), but does not accurately identify other precursor peptides, whose sequences are highly variable and are often not annotated in genomes.

**Figure 2.**
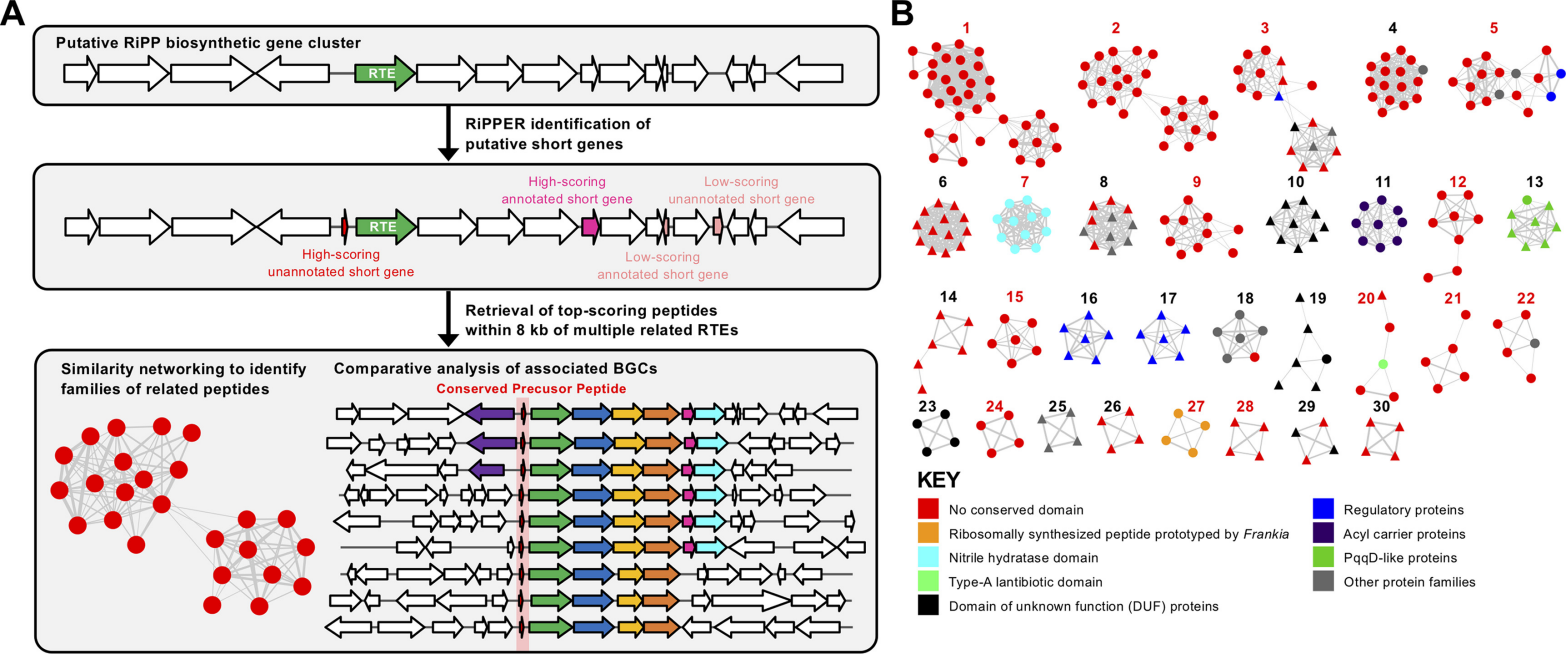
RiPPER identification of putative precursor peptides. (**A**) Schematic of RiPPER workflow where a cluster is identified based on a putative RiPP tailoring enzyme (RTE). (**B**) The 30 largest peptide similarity networks identified using RiPPER for peptides associated with *tfuA*-like genes in Actinobacteria. Red numbers indicate networks predicted to comprise of authentic precursor peptides (see Supplementary Table S1 and Figures S7-S20) and triangular nodes indicate peptides encoded on the opposite strand to the RTE gene. Additional color-coding of nodes reflects domains with a probable association with a biosynthetic gene cluster and includes putative precursor peptides (nitrile hydratase-like (8) and type-A lantibiotic) and other small proteins (PqqD-like proteins (54,55), acyl carrier proteins and regulatory proteins).

To enable the sequence independent discovery of precursor peptides, we sought to identify short ORFs that possess similar genetic features as other genes in a given gene cluster, including ribosome binding sites, codon usage and GC content. Prodigal (PROkaryotic DYnamic programming Gene-finding ALgorithm) uses these criteria to identify bacterial ORFs (39). Therefore, following RODEO retrieval of nucleotide data, we implemented a modified form of this algorithm to specifically search for ORFs that encode for peptides of between 20 and 120 amino acids within apparently non-coding regions near to a predicted RTE (Figure 2A). Given the prevalence of characterized precursor peptides that are encoded on the same strand as a tailoring gene, a same strand score is added (custom parameter; default = 5). A modified GenBank file is generated by RiPPER that annotates these putative short ORFs within the putative BGC (Supplementary Figure S1), and these are ranked alongside annotated short genes based on their Prodigal score. RiPPER then retrieves the top three scoring ORFs within ±8 kb of the RTE, plus any additional high scoring ORFs over a specified score threshold that represent probable genes. These are then assessed for Pfam domains (37) and data associated with each peptide is tabulated for further processing.

To validate this approach, we used RTE accession numbers that had previously been used to identify lasso peptide (13) (RODEO), microviridin (46) and thiopeptide (14) (RODEO) gene clusters. In each case, class-specific rules had been used to identify associated precursor peptides. These RiPP classes are well-suited to method validation as they have diverse gene cluster features and precursor peptide sequences, and span multiple bacterial taxa. In addition, the genes encoding these small peptides are often not annotated in genome sequences (13). We therefore used RiPPER with the same protein accessions as those previous studies to retrieve BGCs and associated precursor peptides. Comparison of the RiPPER outputs with these studies revealed that lasso peptide and microviridin precursor identification was highly reliable. 1056 out of 1122 (94.1%) and 279 out of 288 (96.7%) peptides identified by those prior mining studies were identified by RiPPER (Table 1, Supplementary Datasets 1–2). An analysis of Prodigal scores of these validated precursor peptides showed that this scoring approach is suited to the identification of RiPP precursor peptides (Supplementary Figure S5), despite their small size and the possibility that horizontal gene transfer could influence codon usage bias.

In contrast, RiPPER only retrieved 438 of the 591 (74.1%) thiopeptide precursors previously identified (Table 1, Supplementary Dataset 3). This was possibly due to the comparatively large size of thiopeptide BGCs, which meant that the ±8 kb search window was not suited to a subset of these BGCs. Widening the generic search reduced specificity of the retrieval, so an additional targeted search step was introduced. All short peptides across the entire gene cluster region (default = 35 kb) that were not retrieved by the first search were analyzed for precursor peptide domains using hidden Markov models (HMMs) recently built by Haft *et al.* (38). Any peptides containing a domain were therefore also retrieved. This provided a minor improvement to RiPPER retrieval of lasso precursor peptides but significantly improved thiopeptide precursor peptide retrieval to 549 out of 591 (92.9%) peptides identified by RODEO (14).

This data demonstrated that the RiPPER methodology was applicable to multiple diverse classes of RiPP, but the generic nature of retrieval meant that only between a half and a quarter (depending on RiPP class) of total retrieved peptides were likely to be precursor peptides (Table 1). We therefore generated peptide similarity networks (40) using peptides retrieved from each RiPPER analysis, where peptides with at least 40% identity were connected to each other. Despite the large sequence variance within each RiPP class, this was highly effective at filtering the peptides into networks of likely precursor peptides. For each RiPPER analysis, the largest network (‘network 1’) contained the majority of precursor peptides identified by previous studies (Table 1, Supplementary Figures S2–S4). Unexpectedly, network 1 of the lasso peptide dataset also contained PqqD domain proteins, a conserved feature of lasso peptide pathways that function as RiPP precursor peptide recognition elements (54,55). These peptides were manually filtered by the Pfam domain results; alternatively, a higher identity cut-off for networking would have separated PqqD domains from network 1. In addition, network 2 comprises of 56 *Burkholderia* peptides that are precursors to capistruin lasso peptides (all identified by RODEO). Notably, for each RiPPER analysis, network 1 contained peptides with the expected precursor peptide domain that were not retrieved by either RODEO (13,14) or the bespoke microviridin analysis (46). In total, this provided over 200 new candidate precursor peptides (Table 1), as well as additional networked peptides with no known domains that could feasibly be authentic precursor peptides. The ability of RiPPER to correctly identify a comparable number of precursor peptides to prior targeted methods demonstrates that the combination of rational ORF identification and scoring, Pfam analysis, and peptide similarity networking can identify RiPP precursor peptides with a high degree of accuracy and coverage without any prior knowledge of the RiPP class.

### Identification of thioamidated RiPP BGCs using RiPPER

As a backbone modification, thioamidation potentially has no requirement for specific amino acid side chains, which means that there may be no conserved sequence motifs within precursor peptide substrates. To guide our identification of thioamidated RiPP BGCs, we identified a curated set of 229 TfuA-like proteins in Actinobacteria whose putative BGCs were retrieved using RiPPER, which showed that each TfuA protein was encoded alongside a YcaO protein but their associated gene clusters could be highly variable. RiPPER retrieved 743 peptides (Supplementary Dataset 4) and peptide similarity networking (40% identity cut-off) yielded 74 distinct networks of peptides, where 30 of these networks featured four or more peptides (Figure 2B, Supplementary Figure S6, Supplementary Table S1). MultiGeneBlast (43) was then employed to compare the BGCs corresponding to each network.

**Figure 3.**
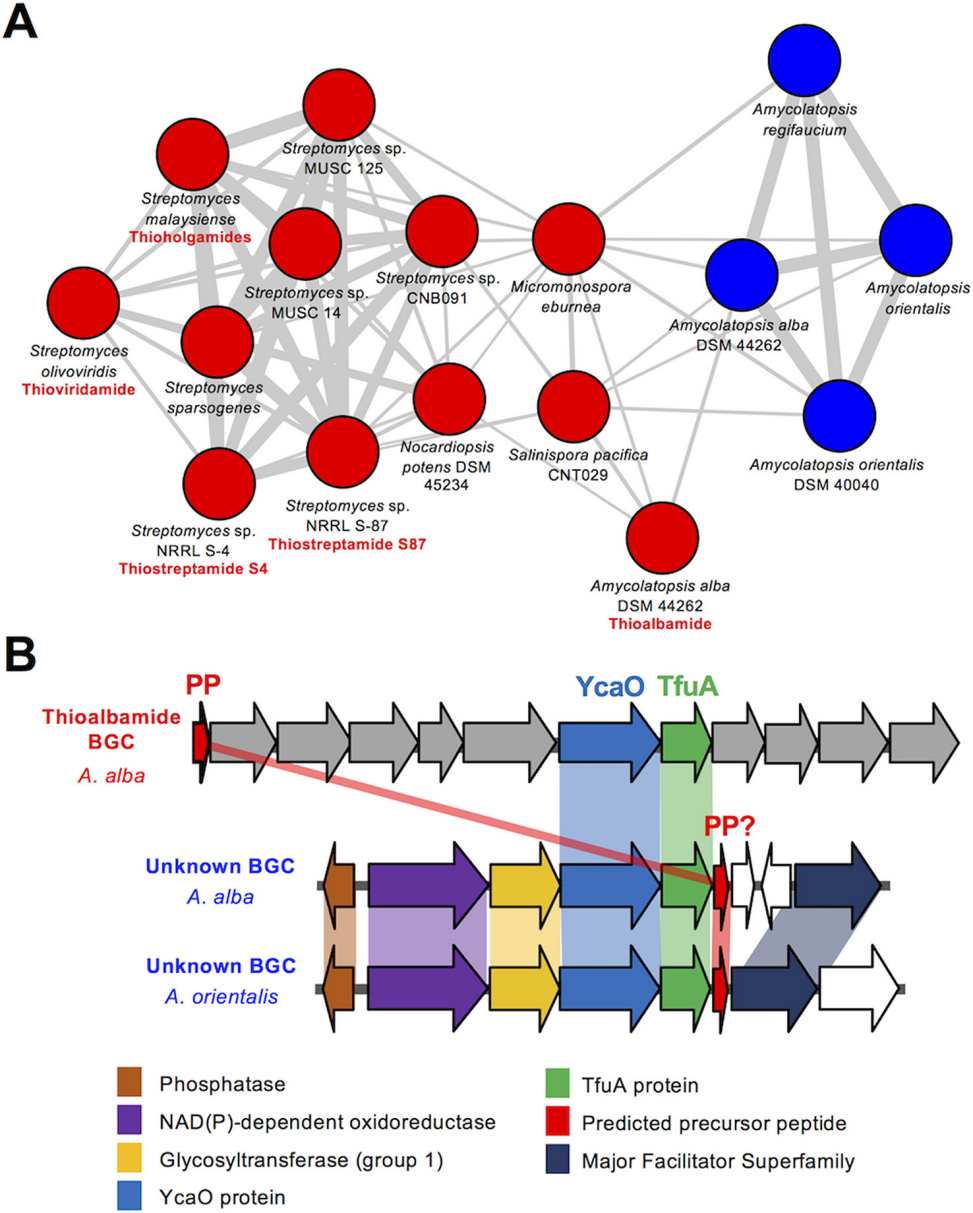
Thioviridamide-like precursor peptides. (**A**) The precursor peptide network that includes both thioviridamide-like precursor peptides (red nodes) and a related but uncharacterized family of precursor peptides from BGCs that are highly different to thioviridamide-like BGCs (blue nodes). Characterized compounds are listed with their respective nodess. (**B**) Comparative analysis of thioviridamide-like and non- thioviridamide-like BGCs from this network where related genes share the same color. See Supplementary Figure S7 for full BGC details.

As an initial proof of concept, this correctly grouped all thioviridamide-like precursor peptides into a single network (Figure 3A). Surprisingly, these precursor peptides were connected with four additional peptides encoded in putative BGCs that are extremely different to thioviridamide-like BGCs; three of these peptides were not previously annotated as genes. These peptides feature extensive sequence similarities with the thioviridamide-like precursor peptides (Supplementary Figure S7), but the BGCs themselves are extremely different, where the only common features with the thioviridamide-like BGCs are the YcaO, TfuA and precursor peptide genes (Figure 3B). More generally, peptide networking guided the identification of a wide variety of probable *tfuA*-containing RiPP BGCs (Supplementary Figures S7–S20). For example, many mycobacteria encode a YcaO-TfuA protein pair, and the largest network of putative precursor peptides is associated with this mycobacterial BGC (Figure 2B, Network 1) where they are usually encoded near a Type III polyketide synthase (PKS) and a sulfotransferase (Supplementary Figure S8). Network 2 consists of 25 related *Streptomyces* peptides that possess high Prodigal scores and are encoded at the start of a conserved biosynthetic operon (Supplementary Figure S9). This is a strong candidate as an authentic RiPP BGC family, yet only 6 of these 25 short peptides were originally annotated.

### Thioamidated RiPPs are a largely unexplored area of the natural products landscape

**Figure 4.**
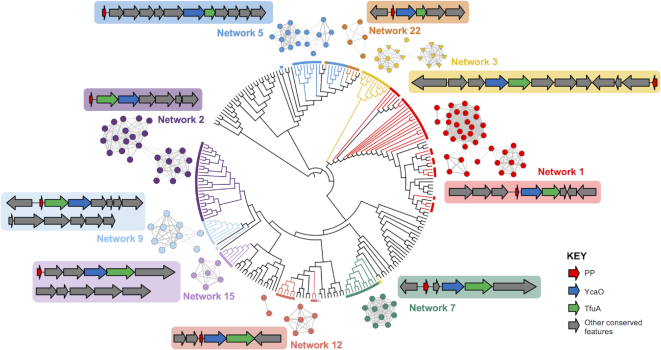
Examples of putative RiPP BGCs and associated TfuA phylogeny. A maximum likelihood tree (branch lengths removed) of TfuA-like proteins is color-coded to indicate the relationship between TfuA-like proteins and the associated networks of putative precursor peptides. Representative BGCs are also shown, where grey genes indicate genetic features that are conserved across multiple BGCs within that family. Fully annotated BGCs are shown in Supplementary Figures S7–S20.

To investigate whether BGC families correlate with the evolutionary relationships of the TfuA proteins, a maximum likelihood tree was constructed from standalone TfuA domain proteins and the peptide networks were mapped to this tree (Figure 4, Supplementary Dataset 5). This showed strong correlations between TfuA phylogeny and precursor peptide similarity. Despite the significant differences between their gene clusters, the thioviridamide-like and non-thioviridamide-like peptides of Network 5 are all associated with closely related TfuA proteins. Unsurprisingly, some TfuA domain proteins are associated with multiple peptide networks due to the abundance of small peptides that are unlikely to be precursor peptides, such as regulatory proteins and RiPP precursor peptide recognition elements (55). For example, almost all peptides from Networks 9, 11 and 18 are associated with the same set of TfuA domain proteins, but Pfam analysis indicates that Networks 11 and 18 consist of acyl carrier proteins and ThiS-like proteins (56), respectively. Therefore, the Network 9 peptides, which are encoded at the beginning of each BGC and feature no conserved domains, are likely precursor peptides for this BGC family (Figure 4).

Pfam analysis indicated that all precursor peptides in Network 7 feature nitrile hydratase domains, which is a common feature amongst precursor peptides across diverse RiPP families (8,57). In total, at least 15 distinct predicted RiPP families were predicted from the top 30 peptide networks (Supplementary Dataset 4, Supplementary Table S1, Supplementary Figures S7–S20), while many smaller networks and singletons are also likely to be authentic precursor peptides, based on their Prodigal scores and positions within BGCs. A comparative analysis with the source GenBank entries indicated that over half of the peptides encoded in these BGCs were not previously annotated (Supplementary Dataset 4). For peptides predicted to be authentic precursor peptides (Supplementary Table S1), unannotated peptides identified by RiPPER were, on average, significantly shorter than annotated peptides (Supplementary Figure S21).

### Characterization of a novel family of TfuA-YcaO BGCs

**Figure 5.**
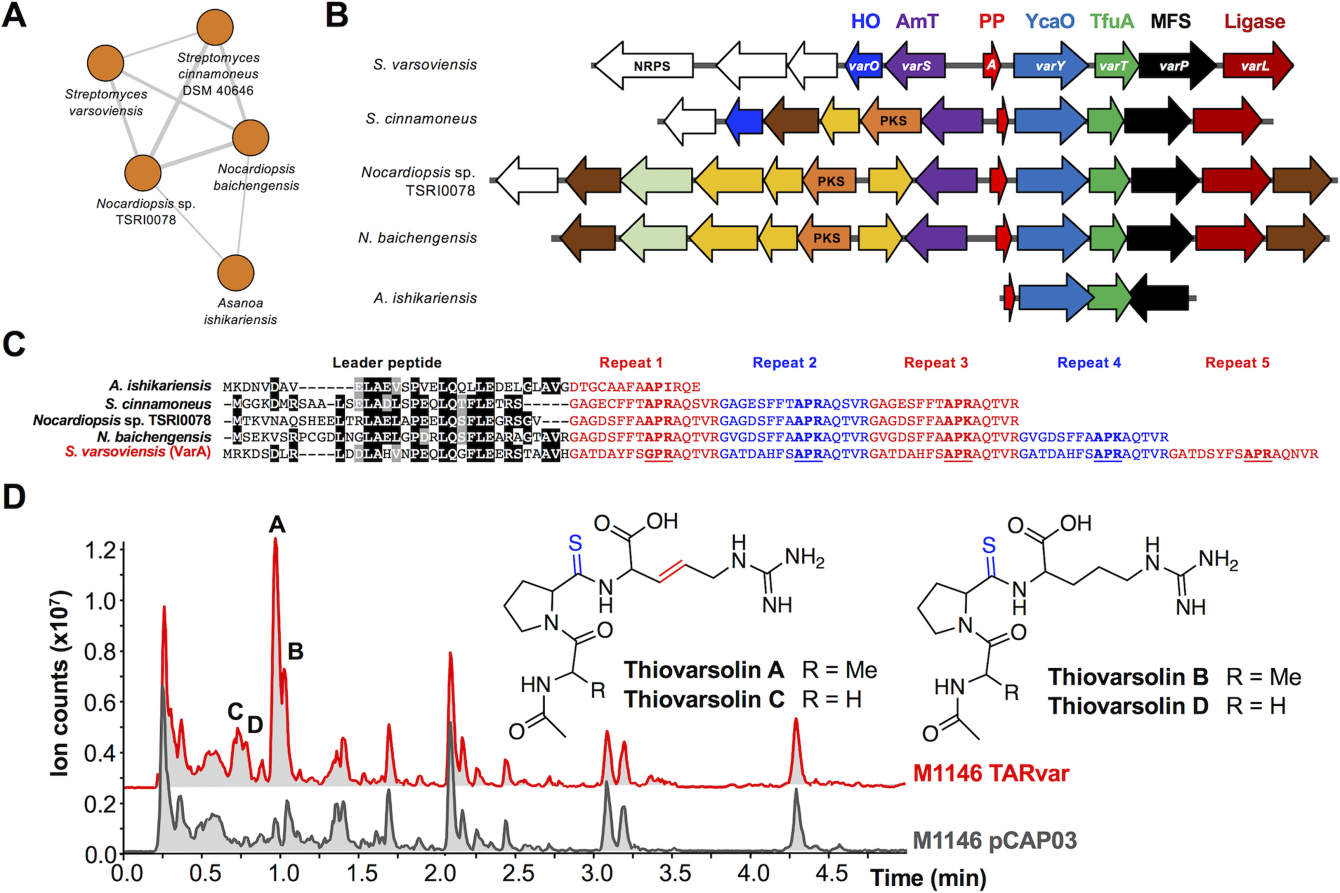
Identification of the thiovarsolin family of RiPPs. (**A**) The associated precursor peptide network. (**B**) BGCs associated with each precursor peptide. The protein product of each *var* gene is listed at the top (HO = heme oxygenase; AmT = amidinotransferase; MFS = major facilitator superfamily) and genes common to multiple BGCs are color-coded by the predicted function of the protein product (see Supplementary Figure S17 for full details). (**C**) Putative repeating precursor peptides identified by similarity networking. The predicted leader peptide is aligned, while the repeat regions are highlighted. Underlined text indicates the partially conserved core peptide that the thiovarsolins derive from, and bold text indicates equivalent residues in the other precursor peptides. (D) Analysis of thiovarsolin production by *S. coelicolor*M1146-TARvar, which contains a 31.7 kb DNA fragment centered on the *S. varsoviensis* BGC. Base peak chromatograms of crude extracts of *S. coelicolor*M1146-TARvar and an empty vector negative control (pCAP03) are shown, with peaks corresponding to thiovarsolins A-D indicated. Thioamidation and dehydrogenation post-translational modifications are highlighted on the thiovarsolin structures.

**Figure 6.**
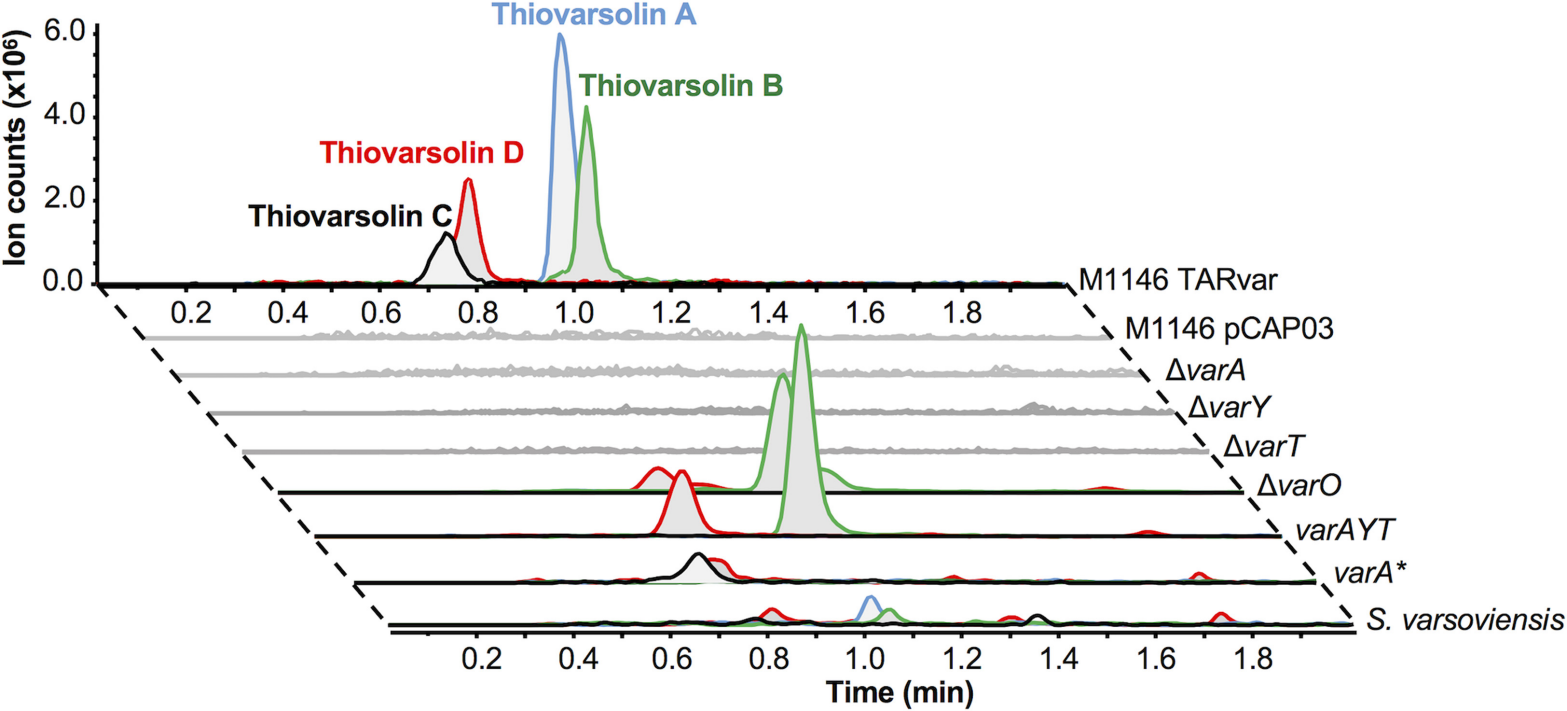
Mutational analysis of thiovarsolin biosynthesis. Extracted ion chromatograms (EICs) are shown for each thiovarsolin (A = *m/z* 399.18, B = *m/z* 401.20, C = *m/z* 385.16, D = *m/z* 387.18). M1146 pCAP03 indicates the empty plasmid control, while each Δ*var* mutation was made in the TARvar construct and expressed in *S. coelicolor* M1146. See text and Supplementary Figure S36 for details of varA*.

To determine whether the newly identified YcaO-TfuA BGCs actually produce thioamidated RiPPs, we focused on Network 22 (Figure 5A), a group of five orphan BGCs with multiple unusual features (Figure 5B). Most notably, the predicted precursor peptides feature a series of imperfect repeats that could reflect a repeating core peptide (Figure 5C), where the family varies from a non-repeating precursor peptide (*Asanoa ishikariensis*) to five repeats (*Streptomyces varsoviensis*). In addition, the *Nocardiopsis* and *Streptomyces* BGCs encode two additional conserved proteins, an amidinotransferase (AmT) and an ATP-grasp ligase, which are homologous to proteins in the pheganomycin pathway (58), and are adjacent to genes encoding non-ribosomal peptide synthetases or PKSs (Figure 5B). Efforts to genetically manipulate *S. varsoviensis* and *Nocardiopsis baichengensis* were unsuccessful and we were unsure of the gene cluster boundaries, so transformation-associated recombination (TAR) cloning (49,59) was employed to capture a 31.7 kb DNA fragment comprising 25 genes (Supplementary Table S2) centered around the *ycaO*-*tfuA* core of the *S. varsoviensis* BGC. Two independent positive TAR clones were conjugated into three different host strains: *Streptomyces lividans* TK24 and *Streptomyces coelicolor* M1146 and M1152 (34) and the resulting TARvar exconjugants were fermented in a variety of media. Liquid chromatography–mass spectrometry (LC–MS) analysis revealed two major compounds (*m/z* 399.18 and *m/z* 401.20), and two minor compounds (*m/z* 385.16 and *m/z* 387.18) not present in the negative control strains (Figure 5D). Small amounts of these compounds could be detected when *S. varsoviensis* was fermented for 10 days (Figure 6, Supplementary Figure S22).

To associate the production of these new compounds to the cloned DNA fragment, PCR-targeting mutagenesis (50) was employed to generate a series of deletion mutants on the putative BGC. A progressive trimming process determined that a cluster of seven genes that are mostly conserved across the *Nocardiopsis* and *Streptomyces* BGCs was sufficient for compound production: *varA* (encoding the predicted repeating precursor peptide), *varY* (the YcaO protein), *varT* (the TfuA protein), *varO* (a heme oxygenase-like protein (60)), *varL* (an ATP-grasp ligase), *varP* (a major facilitator superfamily transporter) and *varS* (an amidinotransferase). The deletion of *varA, varY* and *varT* completely abolished the production of the four new compounds, while the Δ*varO* mutant produced only *m/z* 401.20 and *m/z* 387.18, suggesting that VarO may function as a dehydrogenase (Figure 6). Deletion of *varL, varP* and *varS* did not affect production, despite their conservation in related BGCs (Figure 5B). Δ*varY*, Δ*varT* and Δ*varO* mutants were successfully complemented by expressing these genes under the control of the ermE* promoter, whereas complementation of Δ*varA* required its native promoter. As expected, expression of a 3.7 kb DNA fragment including only *varA*, *varY* and *varT* in *S. coelicolor* M1146 led to the production of *m/z* 401.20 and *m/z* 387.18 (Figure 6, *varAYT*). Collectively, this data show that *varAYTO* are the only genes required for the biosynthesis of this new group of RiPPs, thiovarsolins A–D (observed *m/z* 399.1818, 401.1968, 385.1652 and 387.1808, respectively, Supplementary Table S5).

### The thiovarsolins are thioamidated peptides that derive from the repetitive core of the precursor peptide

The structures of thiovarsolins A and B were determined by NMR (^1^H, ^13^C, COSY, HSQC and HMBC; Supplementary Figures S23-S34, Supplementary Table S6) following large scale fermentation and purification of each compound. This analysis showed that thiovarsolins A and B are *N*-acetylated APR tripeptides in which the amide bond between Pro and Arg is substituted by a thioamide (δ_C_ = 200 ppm) (Figure 5D). This was supported by accurate mass data (Supplementary Table S5) and an absorbance maximum at ∼270 nm for both molecules, which is characteristic of a thioamide group (61). Additionally, a trans double bond is present between Cβ and Cγ of the arginine side chain in thiovarsolin A. This peptide backbone is fully compatible with an APR sequence within the repeats of VarA (Figure 5C). The name thiovarsolin corresponds to linear thioamidated peptides made by *S. varsoviensis*.

Tandem MS (MS^2^) analysis of the thiovarsolins (Supplementary Figure S35) revealed a clear structural relationship between thiovarsolins A (*m/z* 399.18) and C (*m/z* 385.16), as well as between thiovarsolins B (*m/z* 401.20) and D (*m/z* 387.18), which suggested that each 14 Da mass difference could be due to one methyl group. Interestingly, the first repetition of the putative modular core peptide features a GPR motif instead of APR, which could potentially explain this 14 Da mass difference, as well as their observed abundances in relation to thiovarsolins A and B. To test this hypothesis, a mutated version of *varA* was constructed (varA*, Supplementary Figure S36) in which the Ala residue in each repeat was substituted by Gly. This was expressed in M1146-TARvar Δ*varA* using a pGP9-based expression plasmid (62). The resulting strain was only able to produce thiovarsolins C and D (Figure 6, varA*), confirming that these two minor compounds derive from a GPR core peptide. Such an extensively repeating precursor peptide is rare, but is comparable to the variable repeats found in precursor peptides for some cyanobactins (63) and the fungal RiPP phomopsin (64).

Our genetic and chemical analysis of the *var* BGC strongly suggests that the YcaO (VarY) and TfuA (VarT) proteins cooperate to introduce a thioamide bond. Given the absence of a specific protease in the gene cluster, it is plausible that endogenous peptidases are responsible for the liberation of the non-degradable thioamidated APR and GPR tripeptides, which later undergo an *N*-terminal acetylation catalyzed by an endogenous *N*-acetyltransferase, as previously reported for other metabolites containing primary amines (65,66). The timing of VarO-catalyzed dehydrogenation is unclear and could happen directly on the precursor peptide or after proteolysis. Small amounts of thiovarsolins A and B are produced by *S. varsoviensis*, but the lack of a function for *varS* and *varL* suggests that the described thiovarsolins might not be the final products of these pathways. However, no further thiovarsolin-related metabolites could be detected in either *S. varsoviensis* or *S. coelicolor* M1146-TARvar when analyzed by comparative metabolomics and by assessment of MS^2^ data for losses of H_2_S (*m/z* 33.99), which is a fragmentation profile that is characteristic of thioamides (6).

## CONCLUSION

The discovery of the thiovarsolins supports the existence of an unexplored array of thioamidated RiPPs in Actinobacteria. The discovery that a minimal gene set of *varA* (precursor peptide), *varY* (YcaO protein) and *varT* (TfuA protein) is sufficient for the biosynthesis of thiovarsolin B (Figure 6) provides strong evidence that the YcaO-TfuA protein pair catalyze peptide thioamidation in bacteria, which is supported by a parallel study by Mitchell and colleagues on thiopeptide thioamidation (14). It was previously determined that a distantly related pair of homologs catalyze thioamidation of methyl-coenzyme M reductase in archaea (32,33), and that a subset of archaeal YcaO proteins catalyze thioamidation in the absence of a TfuA protein (33). It is therefore possible that there are even more pathways making thioamidated RiPPs than the ones identified in our study, although the closest actinobacterial homologs of the thioamidating TfuA-independent YcaO protein from *Methanopyrus kandleri* (AAM01332.1) are encoded alongside TfuA proteins. Further experimental work is therefore required to determine the breadth of YcaO-domain catalysis and the role of the TfuA partner protein.

The relatively simple thiovarsolin pathway represents a promising system for future biochemical studies of this reaction in the context of RiPP biosynthesis. Unexpectedly, genes conserved across multiple homologous *var*-like pathways (*varS, varP* and *varL*, Figure 5B) were not required for thiovarsolin biosynthesis. Along with *N*-terminal acetylation, this suggests that the identified thiovarsolins may be shunt products, although the production of thiovarsolins by *S. varsoviensis* indicates that they are made naturally, so production is not simply a consequence of heterologous pathway expression. The introduction of a double bond in the arginine residue side chain of the thiovarsolins by VarO would represent new RiPP biochemistry, as heme oxygenases have never been associated with RiPP biosynthesis. This shows that the breadth and diversity of RiPP post-translational modifications is still expanding, which has also been highlighted by recent discoveries of radical SAM enzyme-catalyzed epimerization (57), cyclization (67,68) and β-amino acid formation (69) in RiPP pathways.

RiPPER is a flexible prediction tool that can be applied to any class of predicted RiPP tailoring enzyme to aid in the discovery of this metabolic dark matter. This more general approach complements existing genome-mining tools such as BAGEL (10), RODEO (13,14), PRISM (70) and antiSMASH (12), which all provide in-depth analyses and product predictions for established RiPP families. The underlying logic of RiPPER differs significantly to BAGEL4, antiSMASH 4.0 (which incorporates RODEO) and PRISM 3, which all identify gene clusters based on sets of conserved protein domains predicted to be involved in biosynthesis. With these tools, if established RiPP gene cluster families are identified, predicted precursor peptides and modifications are sometimes displayed. In contrast, the user dictates the gene clusters searched in RiPPER, which aids in the identification of precursor peptides, and this is most effective when multiple similar gene clusters are analyzed in parallel (e.g. Figure 2B). This difference in operation and output makes it difficult to make meaningful comparisons between tools.

The *de novo* identification of precursors to lasso peptides, microviridins and thiopeptides highlights the scope of RiPPER, which was achieved without any specific rules for these RiPP families. The methodology proved to be highly adept at identifying previously overlooked precursor peptide genes, and the method parameters can be easily adapted based on prior knowledge of a given RiPP family (min/max gene length, max distance from RTE, same strand score and peptide score threshold, for example). In our TfuA analysis, peptide networking proved to be a highly effective method to prioritize related precursor peptides and their associated BGCs for further analysis, where it highlighted the existence of likely RiPP families as opposed to the coincidental presence of a small ORF near a putative BGC. The diversity of TfuA-associated precursor peptides identified in Actinobacteria highlights the utility of a generic precursor peptide identification tool and provides the basis for investigating the breadth of this RiPP family. It will be fascinating to determine both the structure and function of these cryptic metabolites.

## DATA AVAILABILITY

RiPPER is available at: https://github.com/streptomyces/ripper and https://hub.docker.com/r/streptomyces/ripdock/ Thiovarsolin gene cluster information is available at https://mibig.secondarymetabolites.org (accession number BGC0001849).

## Supplementary Material

Supplementary Data
