# Supplementary material for: Uncovering the unexplored diversity of thioamidated ribosomal peptides in Actinobacteria using the RiPPER genome mining tool
Source: Nucleic Acids Res. 2019 Mar 27;47(9):4624–37. doi: 10.1093/nar/gkz192 (PMC6511847; doi:10.1093/nar/gkz192)
Supplement: Supplementary Data [file gkz192_supplemental_files.zip › Varso_NAR_SI_final.pdf]

SUPPLEMENTARY INFORMATION

Uncovering the unexplored diversity of thioamidated ribosomal peptides in Actinobacteria using the RiPPER genome mining tool

Javier Santos-Aberturas[1], Govind Chandra[1], Luca Frattaruolo[1], Rodney Lacret[1], Thu H. Pham[1], Natalia M. Vior[1], Tom H. Eyles[1] and Andrew W. Truman[1,*]

[1] Department of Molecular Microbiology, John Innes Centre, Norwich, Norfolk, NR4 7UH, UK

* To whom correspondence should be addressed. Tel: +44(0)1603 450750; Email: andrew.truman@jic.ac.uk

SUPPLEMENTARY METHODS

Culture media composition

All amounts given in g L^{-1}, unless otherwise specified:

LB (Lysogeny Broth): 10 tryptone, 5 yeast extract, 10 NaCl, pH 7. **DNB** / **DNAm** (Difco™ Nutrient Broth/Agar medium): purchased from Becton, Dickinson and Company. 20 agar was added for the solid version. **SFM** (Soya Flour Mannitol): 20 soya flour, 20 mannitol, 20 agar. **TSB** (Tryptic Soy Broth): Purchased from Oxoid. **BPM** (Bottromycin Production Medium): 10 glucose, 15 Difco™ soluble starch, 5 yeast extract, 10 soya flour, 5 NaCl, 3 $CaCO_3$. **MI** ("Monkey Island", modified actinonin production medium): 10 glucose, 10 Difco™ soluble starch, 20 corn liquor step, 20 soy flour, 2.5 NH_4Cl, 3 NaCl and 6 $CaCO_3$, pH 6.2. **TM1** (Teicoplanin Medium 1): 30 malt extract, 10 glucose, 15 soybean meal, 5 yeast extract, 4 $CaCO_3$. **E25**: 25 dextrose, 4 meat extract, 1 yeast autolysate, 10 soybean meal, 4 peptone, 2.5 NaCl, 5 $CaCO_3$. **GYM**: 4 glucose, 4 yeast extract, 10 malt extract, 2 $CaCO_3$, pH 7.2. **YPDA**: 10 bacto yeast extract, 20 bacto peptone, 20 glucose monohydrate, 40 mg L^{-1} adenine hemisulfate. 20 agar were added for the solid version. **SD+CSM-Trp**: 1.7 YNB-AA-$(NH_4)_2SO_4$ (Formedium), 5 $(NH_4)_2SO_4$, 20 glucose, 20 agar, 20 adenine, and 740 mg L^{-1} CSM-Trp (Formedium).

Statistical analysis of predicted precursor peptides

The RiPPER output indicates whether a predicted peptide-coding ORF was already annotated in the original GenBank file. Data for predicted precursors peptides in BGCs identified from the

top 30 networks (Networks 1, 2, 3, 5, 7, 9, 12, 15, 20, 21, 22, 24, 26, 27 and 28; Table S1) were retrieved and classified as “Not annotated” (88 peptides) or “Already annotated” (73 peptides) (Figure S21). The statistical significance of the difference in peptide length distributions between these sample sets was assessed using the two-tailed Mann-Whitney *U* test, which provided a *p*-value of <0.0001 (Z-score = -9.04956).

Construction of a minimal thiovarsolin gene cluster (pIJ10257-*varA*p*YT*)

High-fidelity PCR amplification (primers CP3 and CP8) was used to obtain a 3.7 kb DNA fragment containing *varA* (including its putative promoter region), *varY* and *varT*. The gel-purified PCR product was digested with NdeI and HindIII, and cloned by ligation into NdeI - HindIII digested pIJ10257. The resulting construct (pIJ10257-varAYT) was introduced into *S. coelicolor* M1146 by intergeneric conjugation and selection with nalidixic acid (25 µg mL^{-1}) and hygromycin (50 µg mL^{-1}).

Site-directed mutagenesis of *varA*

The *varA* gene was mutated to generate a version that encodes a peptide where the four repetitions containing APR were replaced by repetitions containing GPR, and residues flanking this motif were mutated to be identical to the natural GPR-containing repeat (peptide VarA*, Figure S36). The presence of highly repetitive sequences and high G+C content meant that commercial gene synthesis failed. Therefore, the construction of the *varA** mutant was achieved by a series of primer extension PCR reactions employing codon degenerated primers to avoid undesired annealing in the repetitive core region of *varA*. To achieve multiple mutations across such a highly repetitive peptide, this method necessitated the introduction of two additional mutations within the third repeat region (Figure S36).

An initial DNA fragment (including the *varA* promoter region, the leader peptide and the first 2.5 mutant repetitions of the core peptide) was generated by three consecutive PCR extension reactions (first primers: CP3 and AG1; second primers: CP3 and AG2; third primers: CP3 and AG3), which was gel purified, digested with NdeI and XbaI, and ligated into NdeI - XbaI linearized pGP9 (1) to yield pGP9_varA*p_partial. A second DNA fragment (containing the last 2.5 mutated repetitions of the core peptide and the intergenic region following *varA*) was generated by three consecutive PCR extension reactions (first primers: AG4 and CP4; second primers: AG5 and CP4; third primers: AG6 and CP4). This was gel purified, digested with KpnI and Hind III, and cloned into KpnI – HindIII linearized pGP9_*varA**_partial to yield the final construct, pGP9_*varA**p, which was sequenced and introduced into *S. coelicolor* M1146-TARvar Δ*varA*_clean by intergeneric conjugation and selection with nalidixic acid (25 µg mL^{-1}), kanamycin (50 µg mL^{-1}) and apramycin (50 µg mL^{-1}).

Large scale fermentation, isolation and structural elucidation of thiovarsolins A and B

6 x 2 L flasks, each containing 500 mL BPM, were inoculated with 25 mL of *S. coelicolor* M1146-TARvar seed culture grown in TSA (200 mL in a 1 L flask, 72 h) and incubated at 28 °C with shaking at 250 rpm for 10 days. The culture broth was separated from the mycelium by centrifugation to yield a cell-free supernatant (ca. 3 L), which was concentrated to dryness to afford 32.4 g of crude extract. This extract was resuspended in 0.4 L of distilled water and was then fractionated using vacuum liquid chromatography (VLC) on C-18 RP silica gel using a gradient of H_2O:MeOH (100:0 to 0:100). The major thiovarsolin-containing fraction (2.4 g) was subsequently fractionated on a Sephadex LH-20 column using methanol:water (7:3) as the mobile phase. Thiovarsolin-containing fractions were combined and dried to yield 0.78 g. This was subjected to solid phase chromatography using a C-18 cartridge (Discovery DSC-18, 20 mL) with a gradient of water:methanol (100:0 to 70:30). Fractions containing thiovarsolins A and B were further purified by semipreparative HPLC (Phenomenex Luna PFP(2), 210 mm x 10 mm, 5 mm; 3 mL min^{-1}, UV detection at 268 nm) with a linear gradient of CH_3CN/H_2O (+0.1% formic acid) from 5 to 15% CH_3CN over 30 minutes yielding thiovarsolin A (1.3 mg, retention time = 15.2 min) and thiovarsolin B (0.8 mg, retention time = 18.3 min). 1D and 2D NMR spectra were recorded on Bruker Avance 700, 600 and 400 MHz NMR spectrometers operated using Topspin 2.0 software. Spectra were calibrated to the residual solvent signals of DMSO-d_6 with resonances at δ_H 2.50 and δ_C 39.52.

SUPPLEMENTARY FIGURES

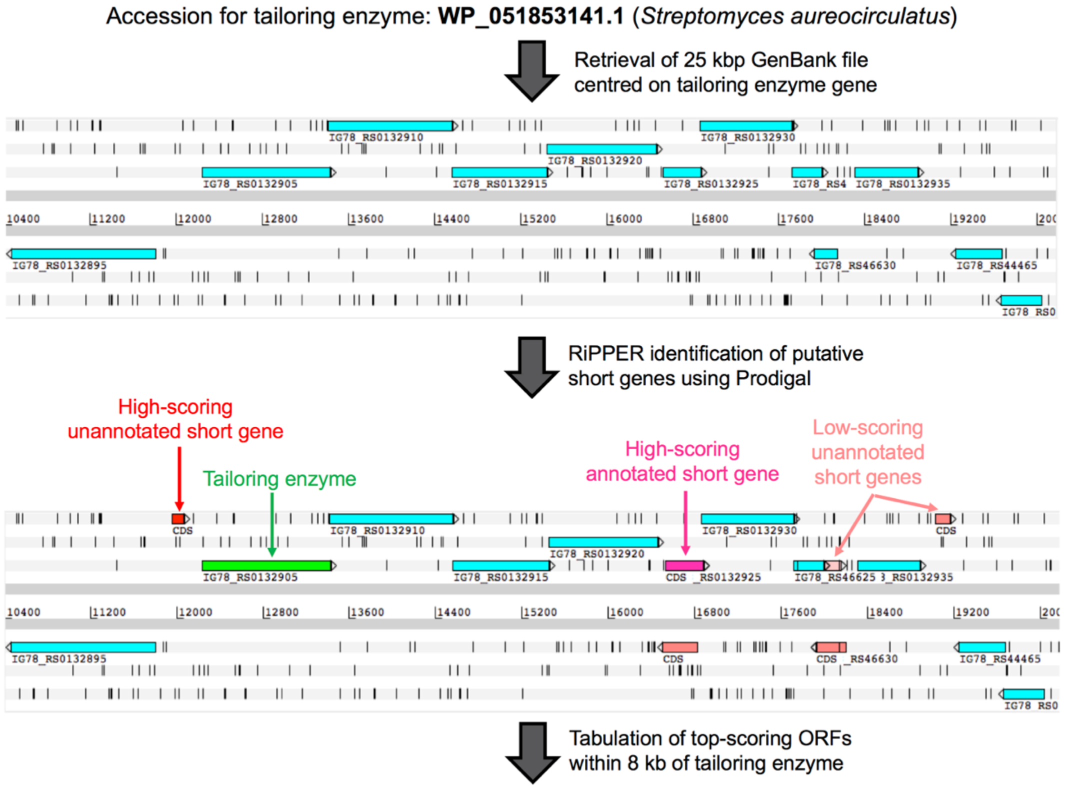

Accession	Organism	PP Serial	FastaID	Sequence	Same Strand	PP_TE Distance	Prodigal score	Pfam
**WP_051853141**	*Streptomyces aureocirculatus*	1	WP_051853141_1	MHASVLSAVEIALTLALVAWMATGFTRLPGRWLRWKMFCRATFTIV TLTGTKDGRTEPVNVYDHLSPGSFILGPPQLQTILNYLTHTGRYDR VDGHGRLLNADGERPIDIRDSRVVL	yes	3100	62.07	none
**WP_051853141**	*Streptomyces aureocirculatus*	2	WP_051853141_2	MVSKSQAAVSTLKPTQETEELTDTVVVAVDVEDEE	yes	174	18.35	none
**WP_051853141**	*Streptomyces aureocirculatus*	3	WP_051853141_3	MLEPSGSNNRGQVPTNVSRLNGRVPARPAKDEPEHYHLDIGYAFT TAHADVGSIQEAEVTGAAWHQLAEAESLVGSRIARAVSTPARTD	no	4501	1.95	none

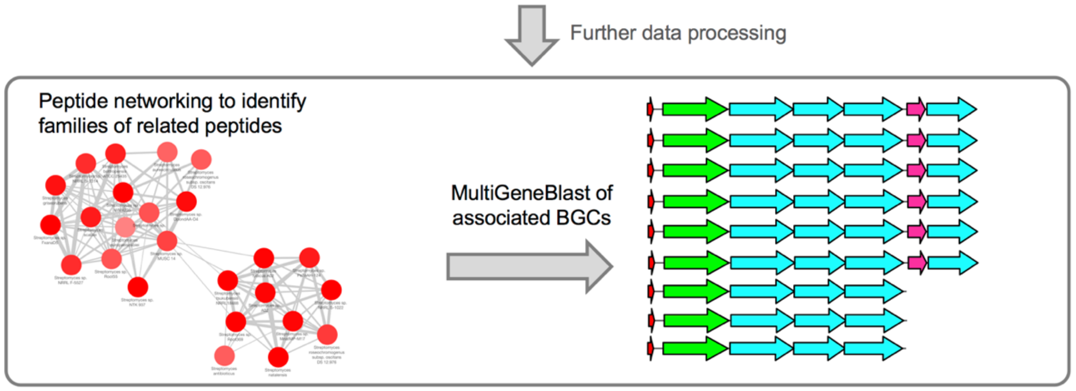

Figure S1. RiPPER workflow showing the output of a single TfuA accession (a short region of the Artemis (2) output is shown), as well as the associated peptide network and MultiGeneBlast (3) analysis (the output is simplified for clarity and simply reflects the Artemis color-coding).

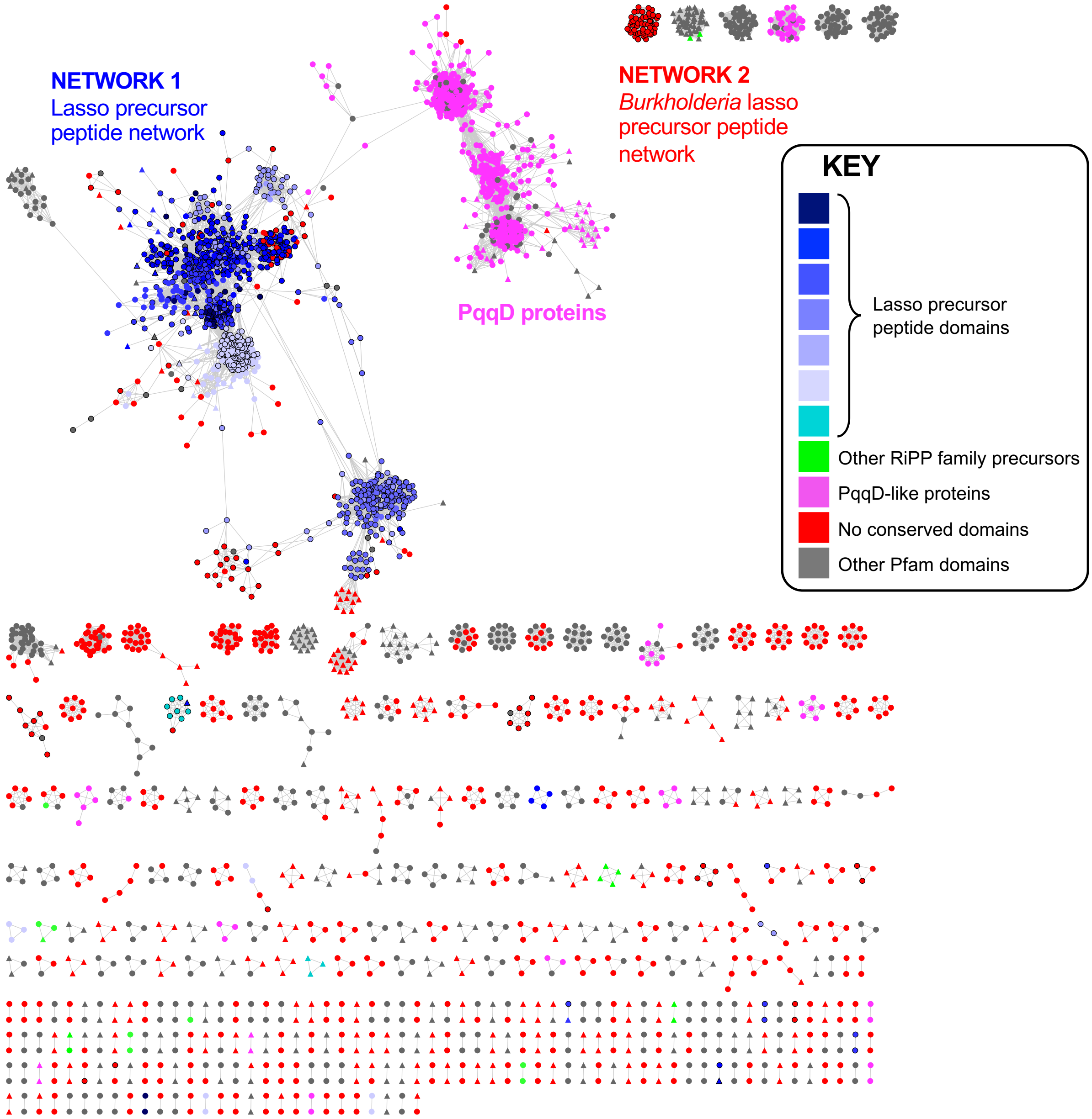

Figure S2. Peptide similarity networks of short peptides identified by RiPPER that are encoded in BGCs of asparagine synthetases previously predicted by RODEO to catalyze lasso peptide formation (4). Each node represents one peptide, have a black border if they match a peptide identified by Tietz *et al*. (4), and are colored by Pfam domain. Putative peptides encoded on the same strand as the asparagine synthetase gene have circular nodes and those on the opposite strain have triangular nodes. An edge cut-off of 40% identity was used.

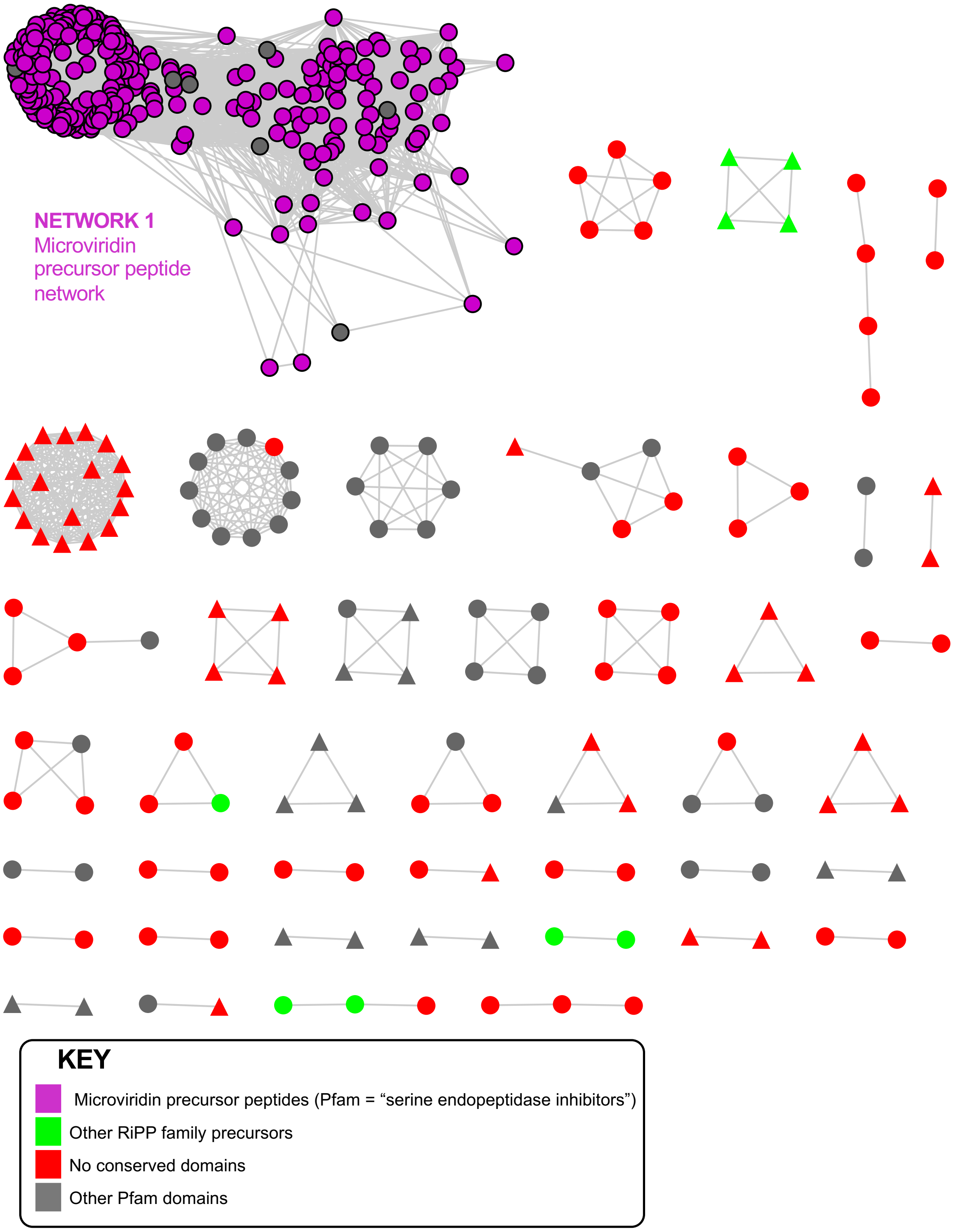

Figure S3. Peptide similarity networks of short peptides identified by RiPPER that are encoded in BGCs encoding homologues of MvdD (or MvdC) that were previously predicted by Ahmed *et al*. to produce microviridins (5). Each node represents one peptide, have a black border if they match a peptide identified by Ahmed *et al*. (5), and are colored by Pfam domain. Putative peptides encoded on the same strand as the *mvdD/C* gene have circular nodes and those on the opposite strain have triangular nodes. An edge cut-off of 40% identity was used.

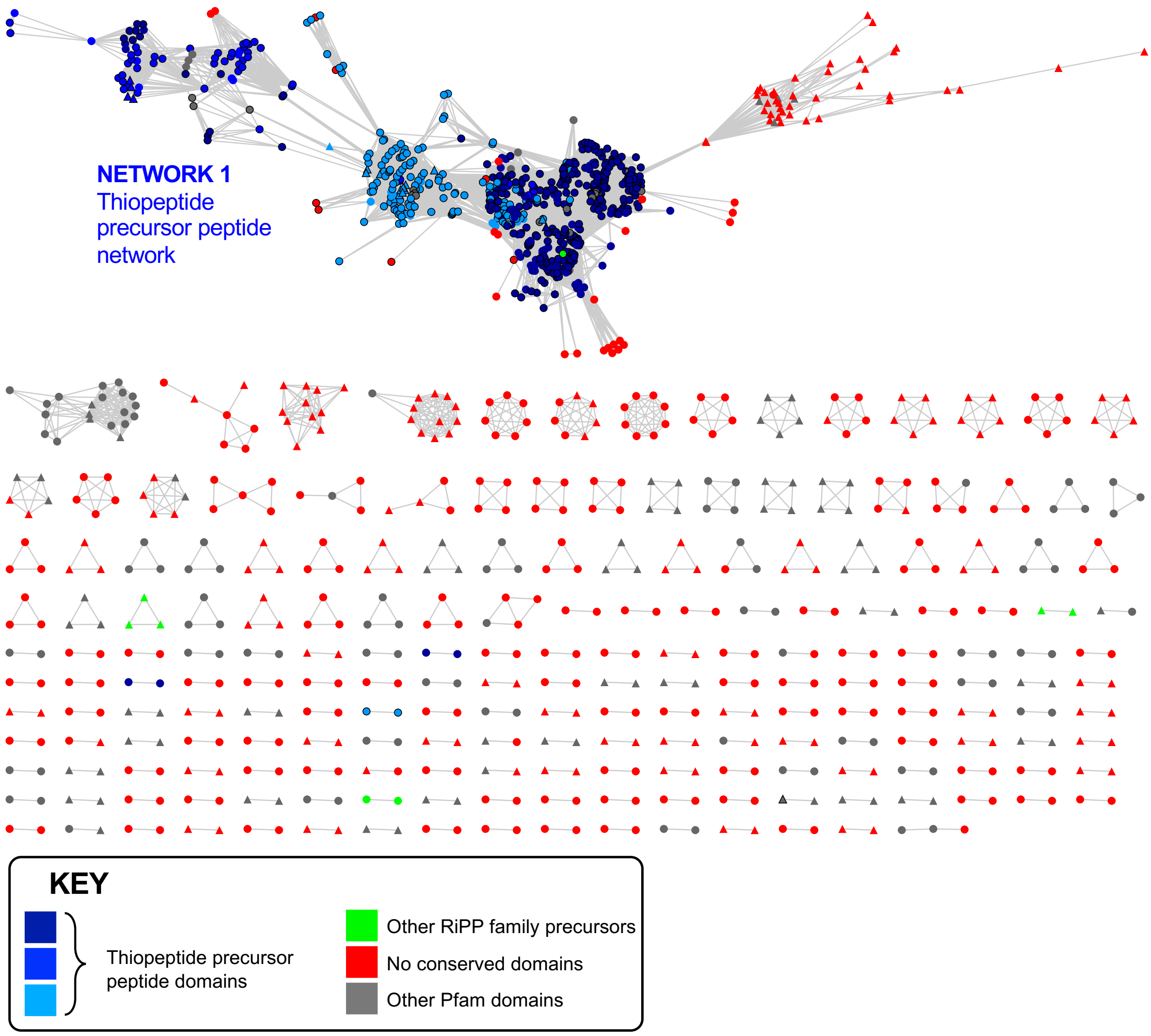

Figure S4. Peptide similarity networks of short peptides identified by RiPPER that are encoded in BGCs encoding [4 + 2]-cycloaddition enzymes that were previously predicted by Schwalen *et al*. to produce thiopeptides (6). Each node represents one peptide, have a black border if they match a peptide identified by Schwalen *et al*. (6), and are colored by Pfam domain. Putative peptides encoded on the same strand as genes encoding [4 + 2]-cycloaddition enzymes have circular nodes and those on the opposite strain have triangular nodes. An edge cut-off of 40% identity was used.

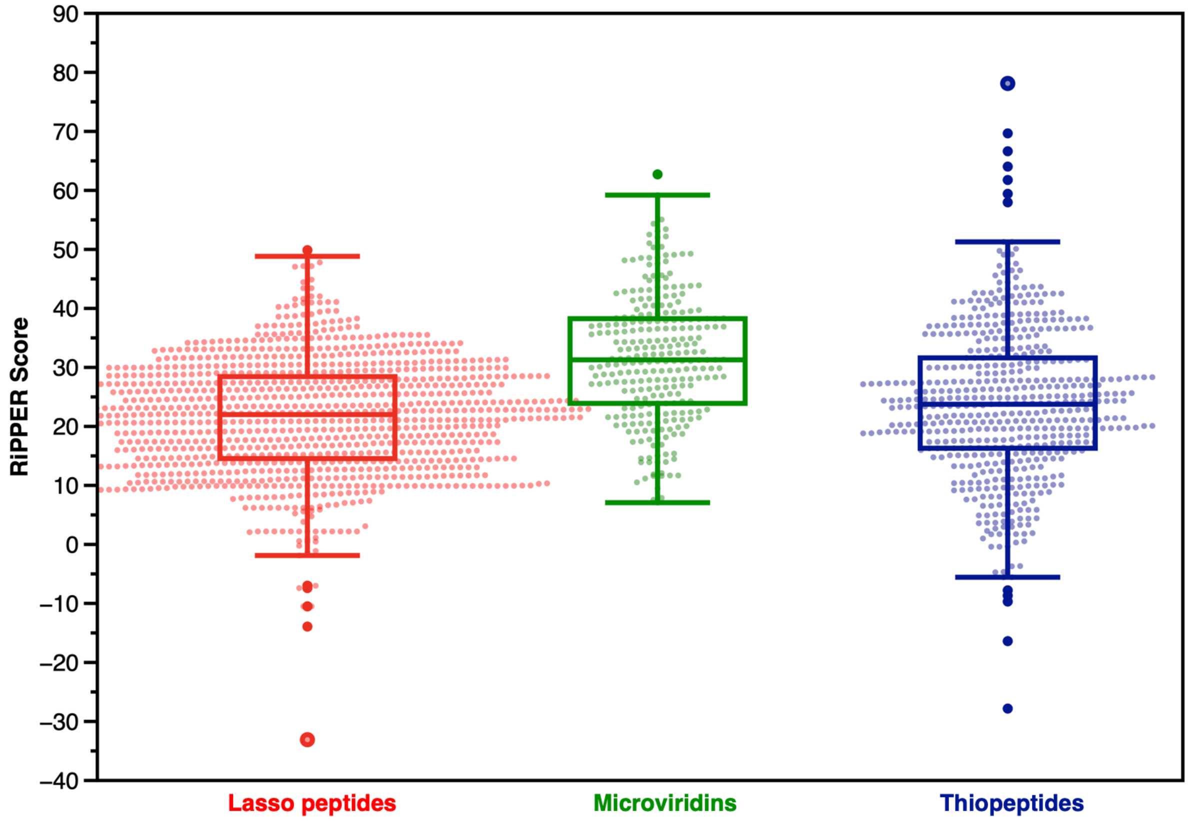

Figure S5. Box plots of the RiPPER scores for the precursor peptides of lasso peptides, microviridins and thiopeptides. Outliers are circled and all raw peptide scores are plotted as dots.

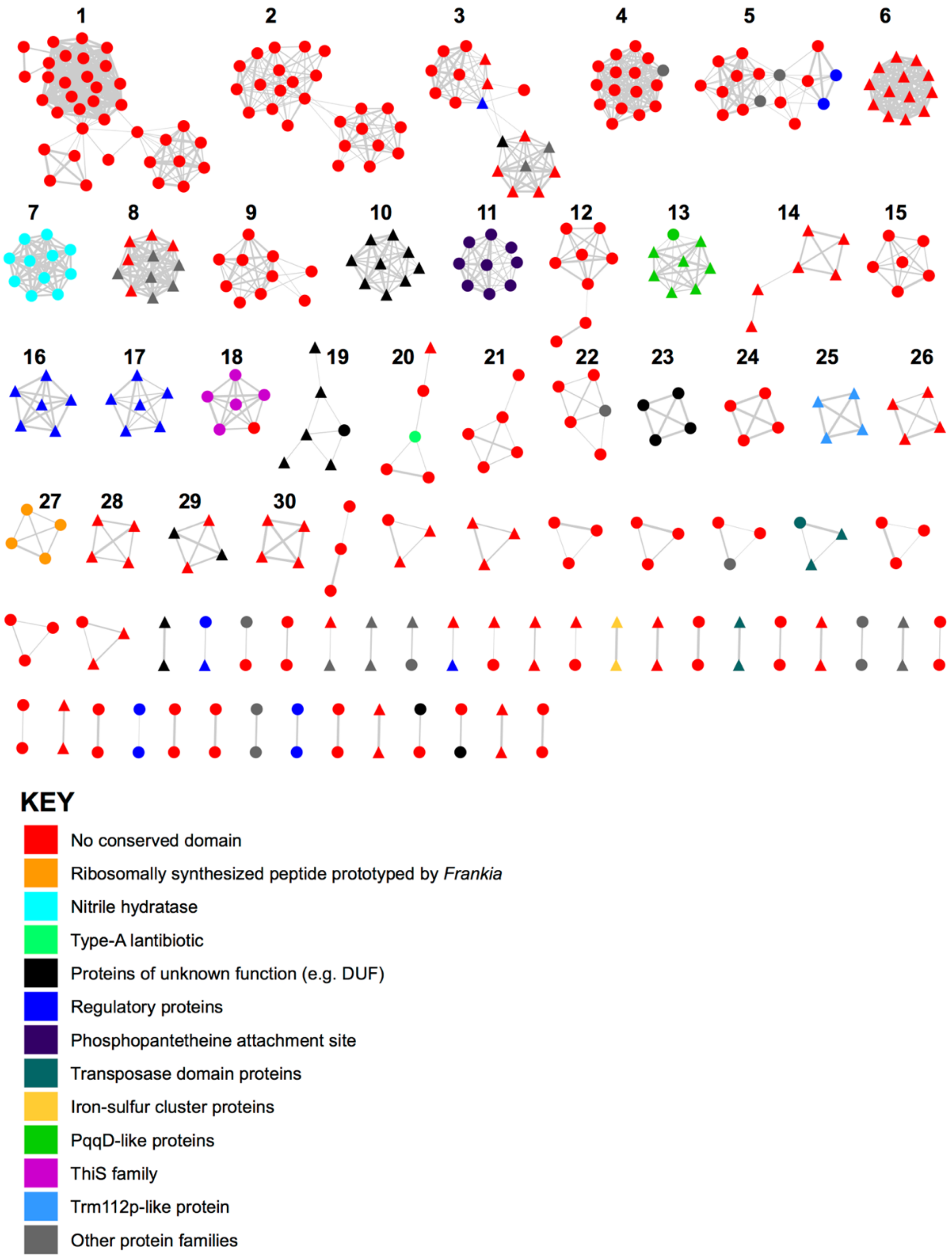

Figure S6. All networks identified for short peptides associated with *tfuA*-like genes using EGN (7) with an identity cut-off of 40%. The top 30 networks (4 or more peptides) are numbered and peptides are color-coded according to Pfam domain. Each node represents a single peptide and are circular if they are on the same strand as the *tfuA*-like gene and triangular if they are on the opposite strand.

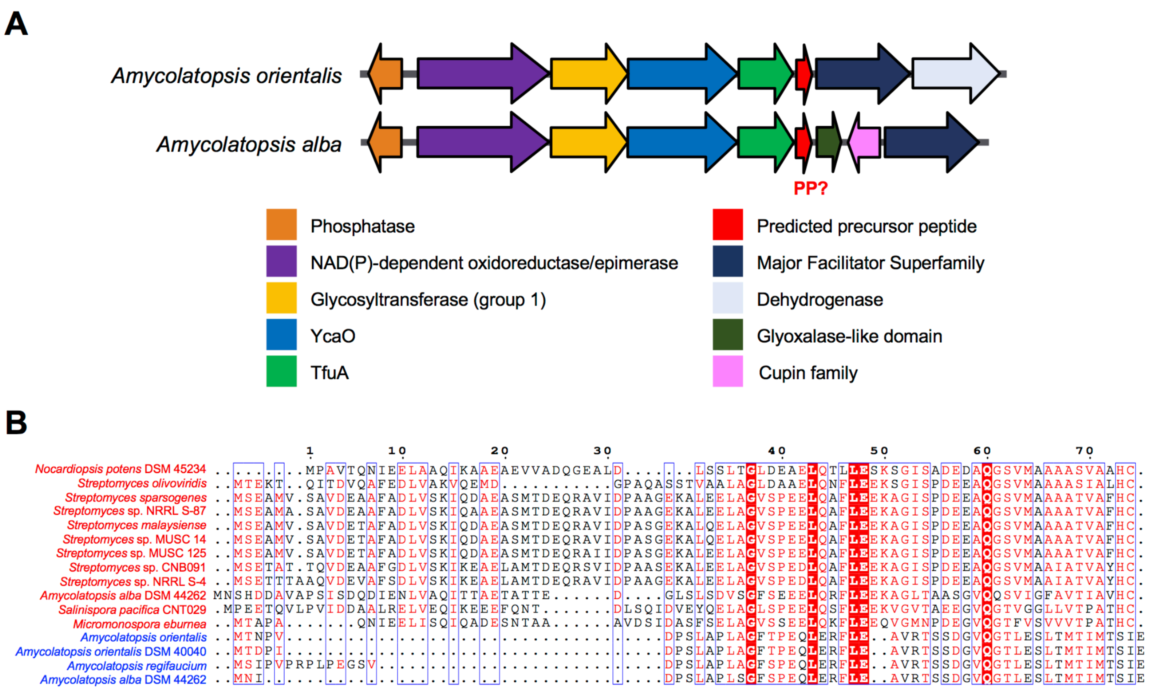

Figure S7. Details of the thioviridamide-containing Network 5. (A) Representative conserved BGCs relating to Fig. 3 in main paper. (B) Alignment of the thioviridamide-like precursor peptides that form Network 5. Peptides with red names indicate peptides with thioviridamide-like features (e.g. terminal HC motif) and BGCs with thioviridamide-like biosynthetic genes, whereas peptides with blue names reflect networked peptides with different putative core sequences and BGCs lacking almost all thioviridamide-like biosynthetic genes. See Frattaruolo *et al.* (8) for details of thioviridamide-lik BGCs. All alignment images were generated using Espript 3.0 (9).

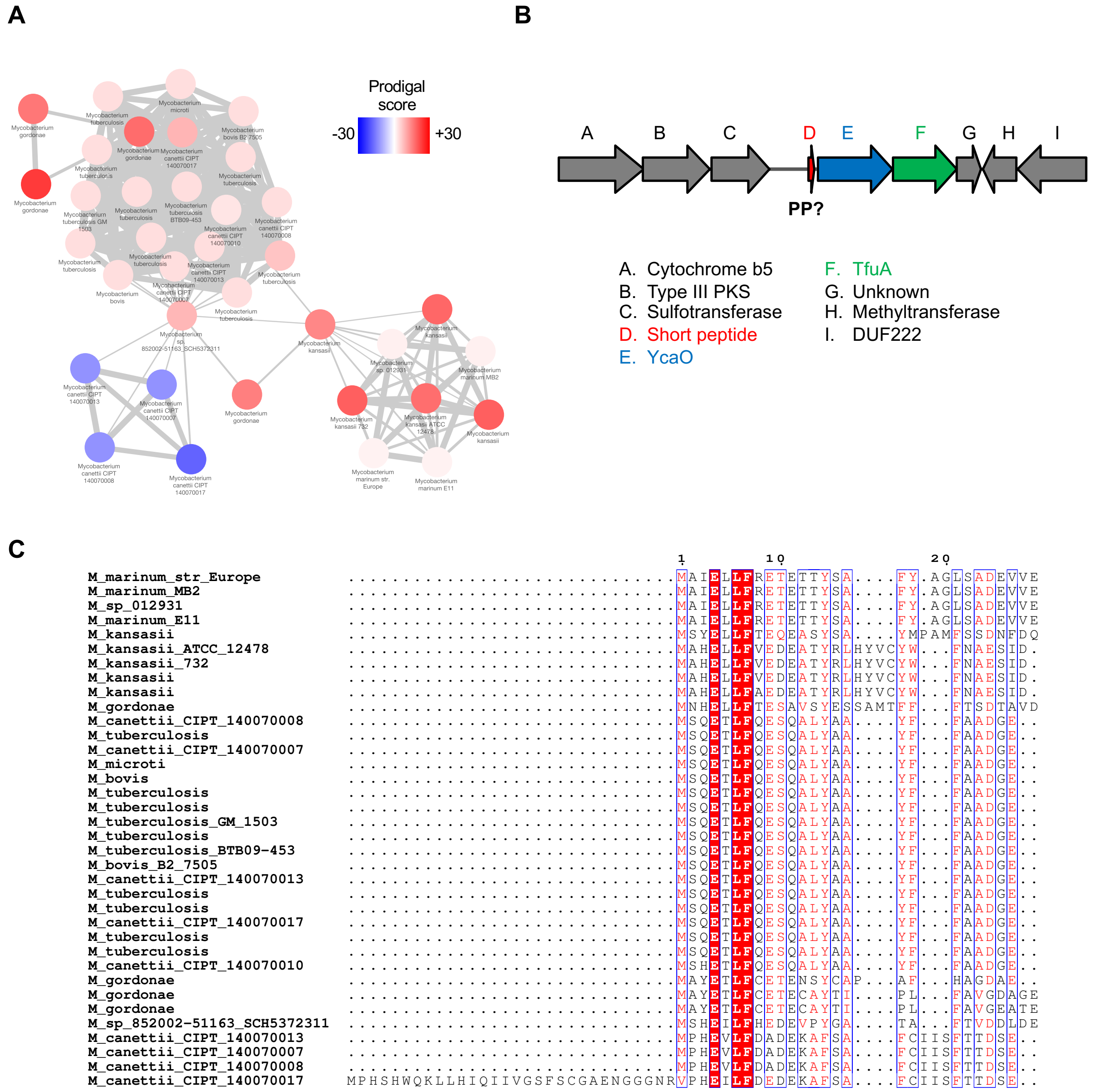

Figure S8. Network 1 overview. (A) Peptide network color-coded by Prodigal score (scores over 30 are all colored red). (B) Representative conserved BGC with PP, YcaO and TfuA genes highlighted. (C) Sequence alignment of all peptides present in network. A red background indicates full conservation, blue boxes represent positions with over 60% sequence equivalence, with the corresponding similar amino acids colored red.

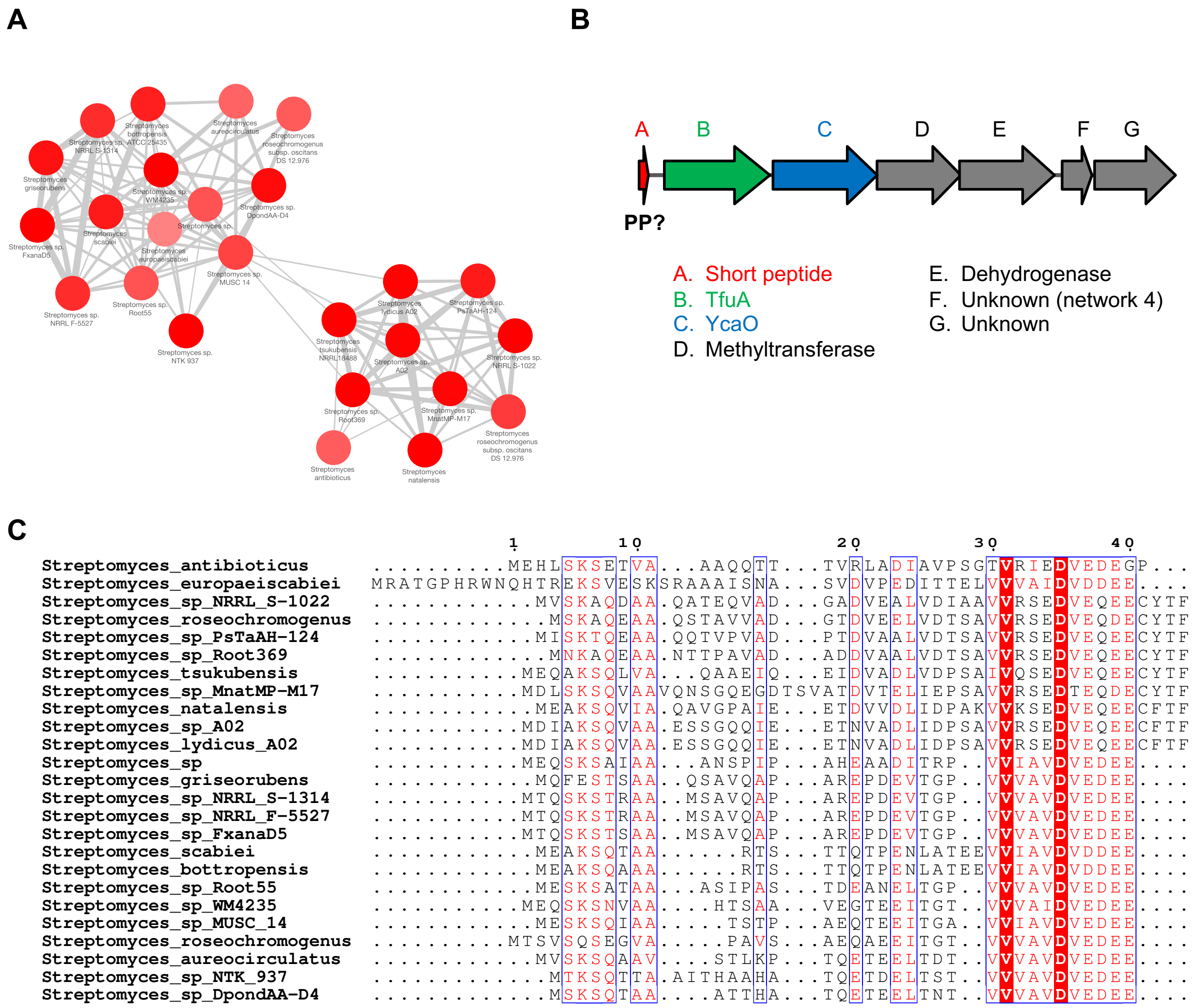

Figure S9. Network 2 overview. (A) Peptide network color-coded by Prodigal score. (B) Representative conserved BGC with PP, YcaO and TfuA genes highlighted. (C) Sequence alignment of all peptides present in network. Network and alignment color-coding is identical to Figure S8.

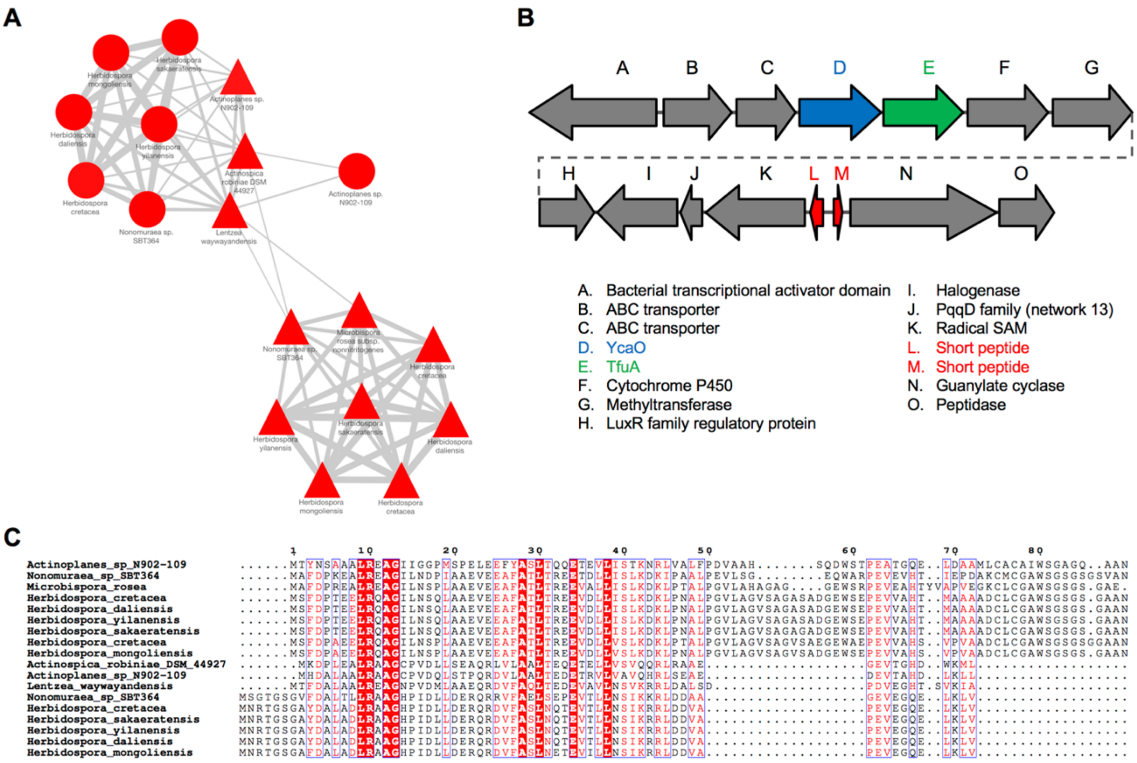

Figure S10. Network 3 overview. (A) Peptide network color-coded by Prodigal score. (B) Representative conserved BGC with PP, YcaO and TfuA genes highlighted; some BGCs only have one short peptide in the network. (C) Sequence alignment of all peptides present in network. Network and alignment color-coding is identical to Figure S8.

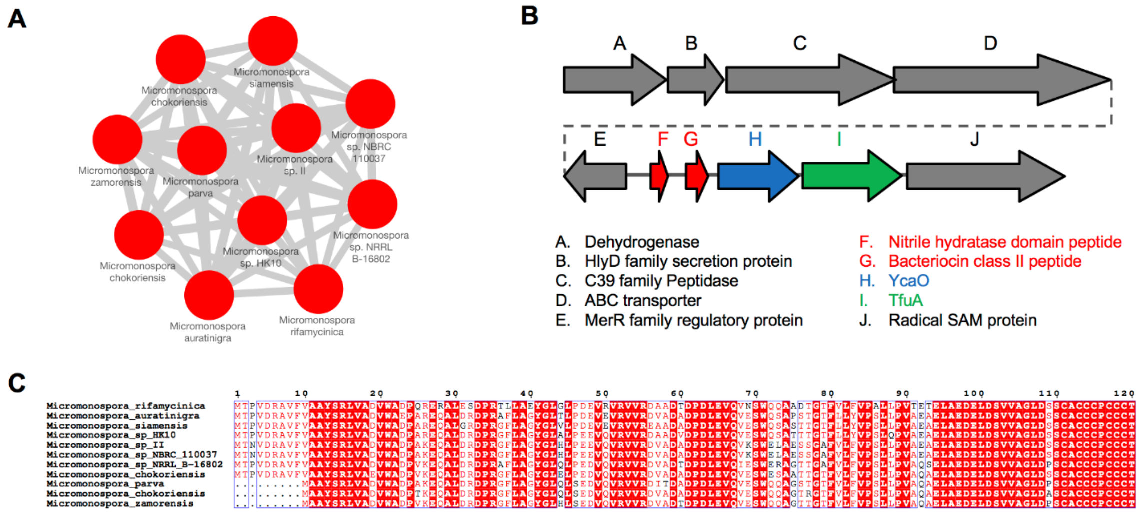

Figure S11. Network 7 overview. (A) Peptide network color-coded by Prodigal score. (B) Representative conserved BGC with PP, YcaO and TfuA genes highlighted. The second PP (gene G) was not identified by RiPPER as the peptide is over 120 AA, but MultiGeneBlast indicates homology to the PPs in this network. (C) Sequence alignment of all peptides present in network. Network and alignment color-coding is identical to Figure S8.

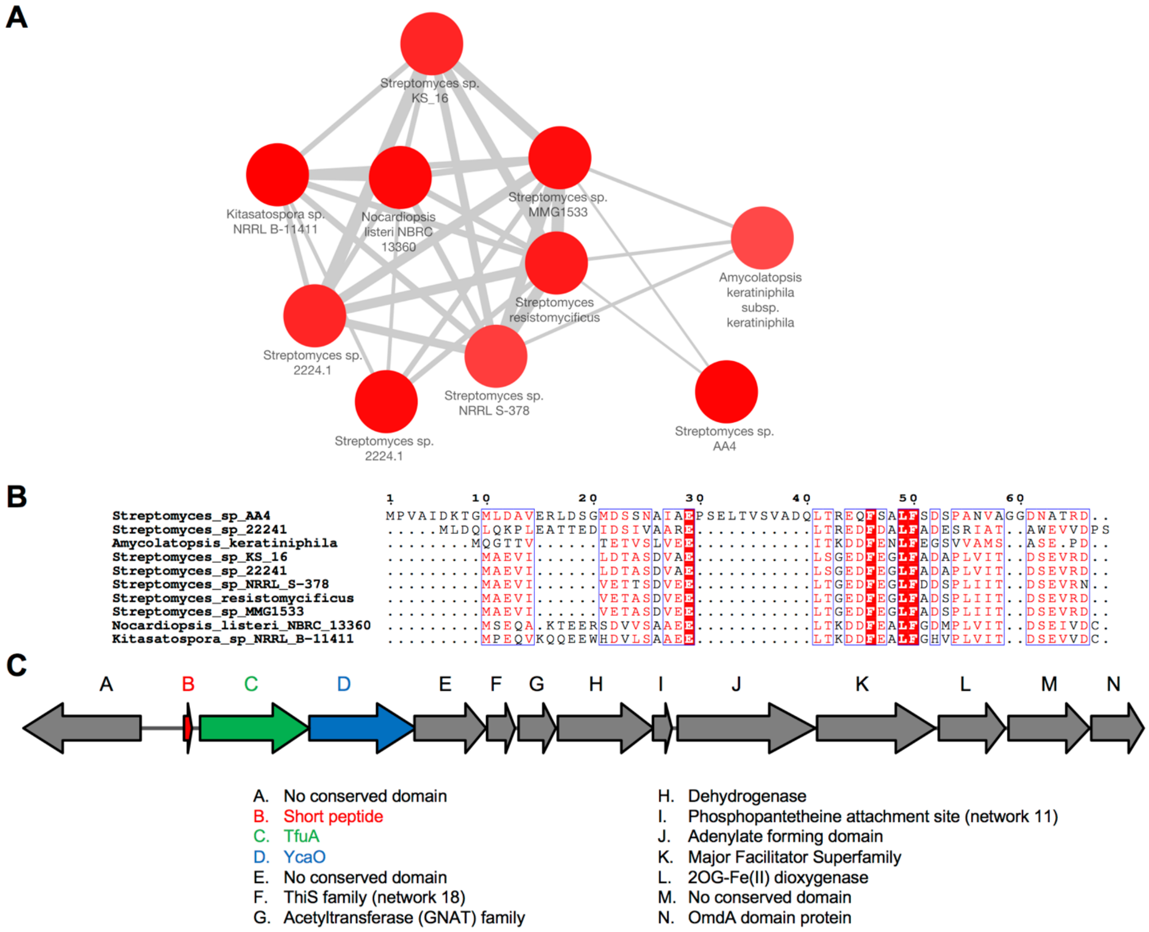

Figure S12. Network 9 overview. (A) Peptide network color-coded by Prodigal score. (B) Sequence alignment of all peptides present in network. (C) Representative conserved BGC with PP, YcaO and TfuA genes highlighted. Network and alignment color-coding is identical to Figure S8.

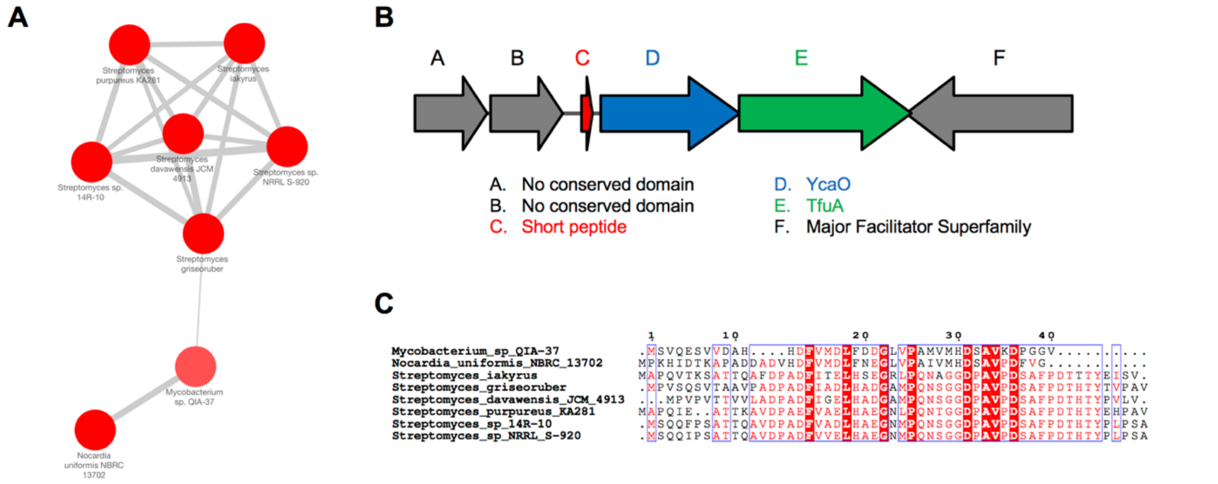

Figure S13. Network 12 overview. (A) Peptide network color-coded by Prodigal score. (B) Representative conserved BGC with PP, YcaO and TfuA genes highlighted. (C) Sequence alignment of all peptides present in network. Network and alignment color-coding is identical to Figure S8.

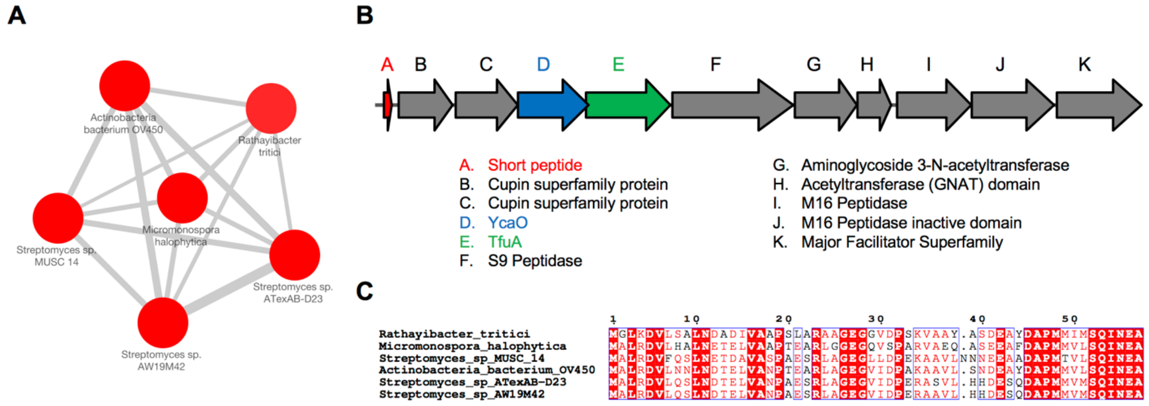

Figure S14. Network 15 overview. (A) Peptide network color-coded by Prodigal score. (B) Representative conserved BGC with PP, YcaO and TfuA genes highlighted. (C) Sequence alignment of all peptides present in network. Network and alignment color-coding is identical to Figure S8.

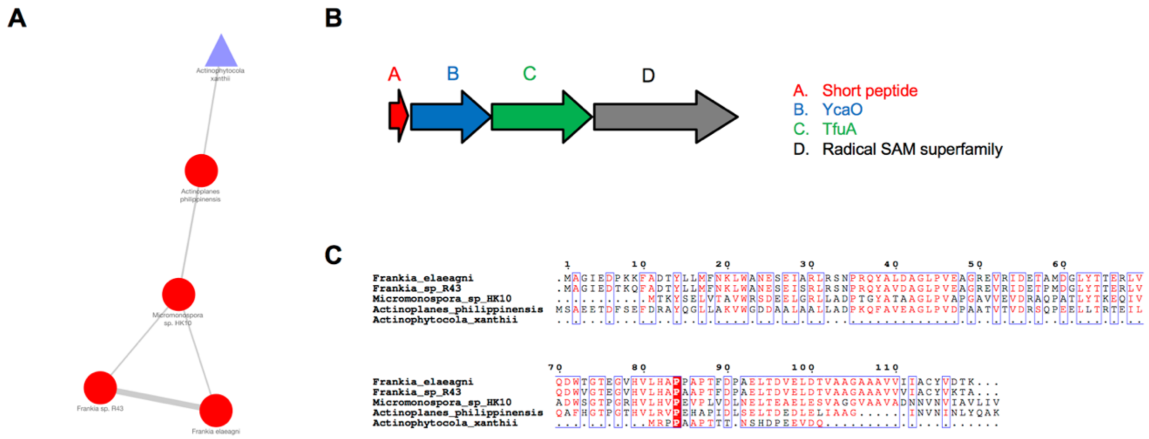

Figure S15. Network 20 overview. (A) Peptide network color-coded by Prodigal score. Low score, different orientation, short length and non-canonical position in relation to putative biosynthetic genes indicates that the *Actinophytocola xanthii* peptide is an outlier. (B) Representative conserved BGC with PP, YcaO and TfuA genes highlighted. (C) Sequence alignment of all peptides present in network. Network and alignment color-coding is identical to Figure S8.

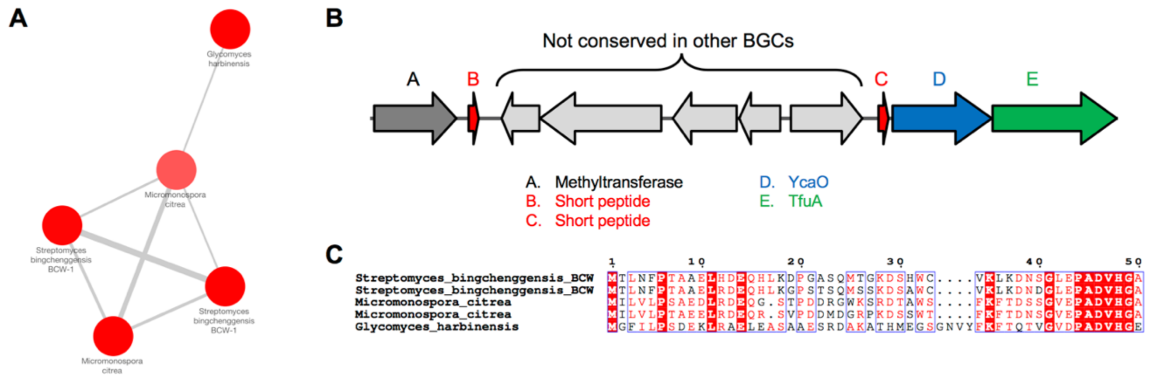

Figure S16. Network 21 overview. (A) Peptide network color-coded by Prodigal score. (B) Representative conserved BGC with PP, YcaO and TfuA genes highlighted. Pale grey genes represent an area between short peptides that is not conserved between gene clusters (*Micromonospora citrea* BGC shown). (C) Sequence alignment of all peptides present in network. Network and alignment color-coding is identical to Figure S8.

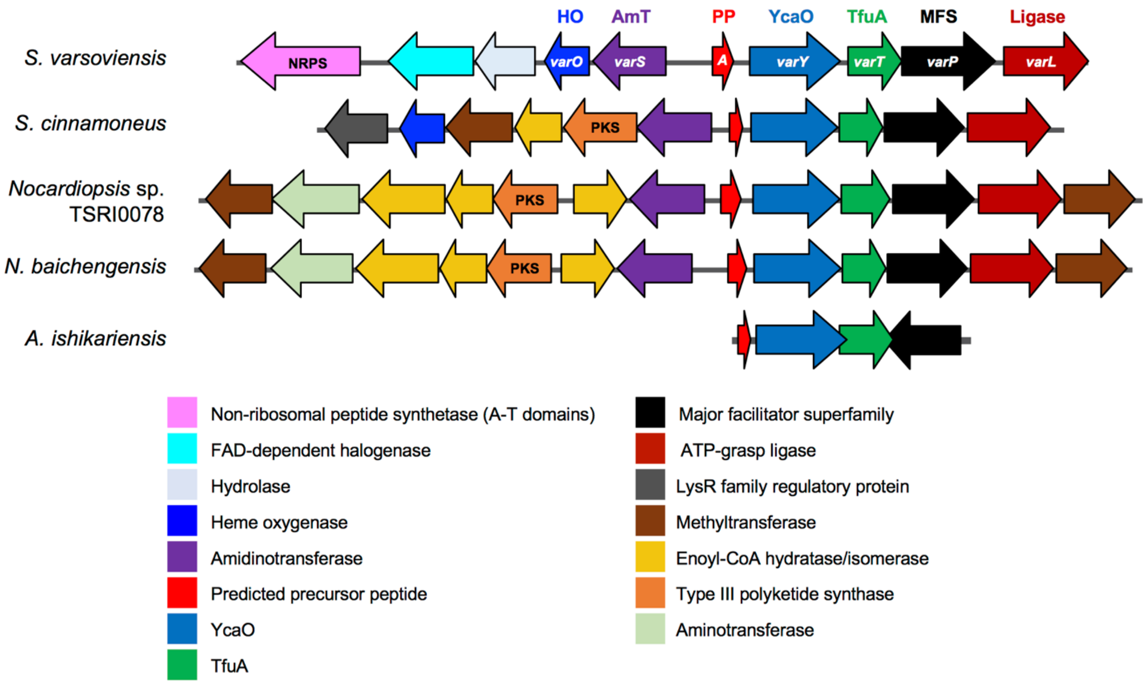

Figure S17. Full annotation of the thiovarsolin-like BGCs (Network 22) shown in Fig. 5B.

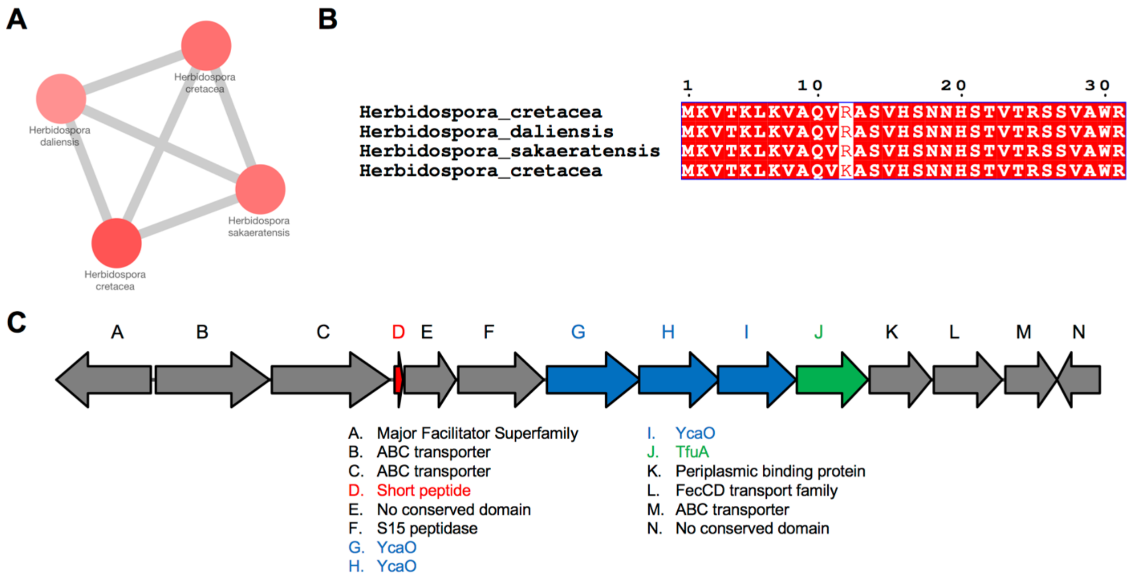

Figure S18. Network 24 overview. (A) Peptide network color-coded by Prodigal score. (B) Sequence alignment of all peptides present in network. (C) Representative conserved BGC with PP, YcaO and TfuA genes highlighted. Network and alignment color-coding is identical to Figure S8.

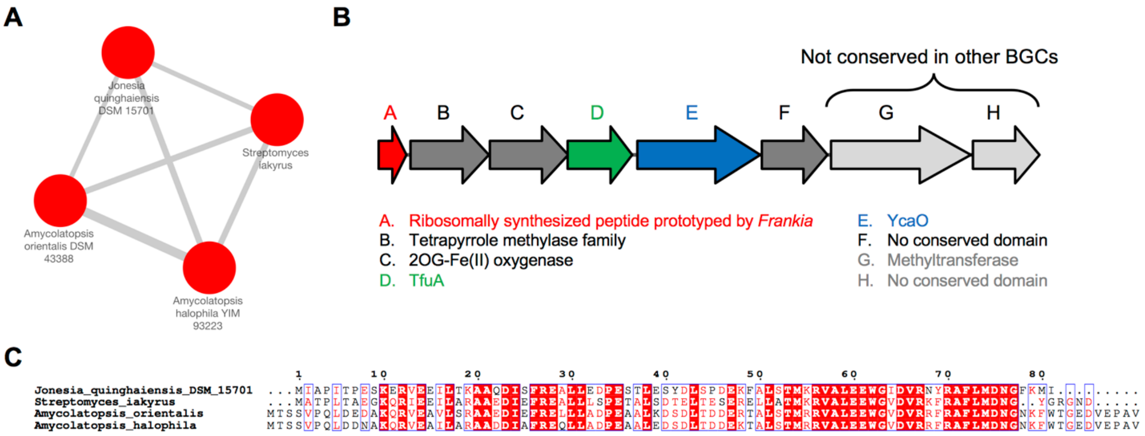

Figure S19. Network 27 overview. (A) Peptide network color-coded by Prodigal score. (B) Representative conserved BGC with PP, YcaO and TfuA genes highlighted. Pale grey genes represent an area that is not conserved between gene clusters (the genes shown are found in the *Amycolatopsis* BGCs, but different genes are in the other BGCs). (C) Sequence alignment of all peptides present in network. Network and alignment color-coding is identical to Figure S8.

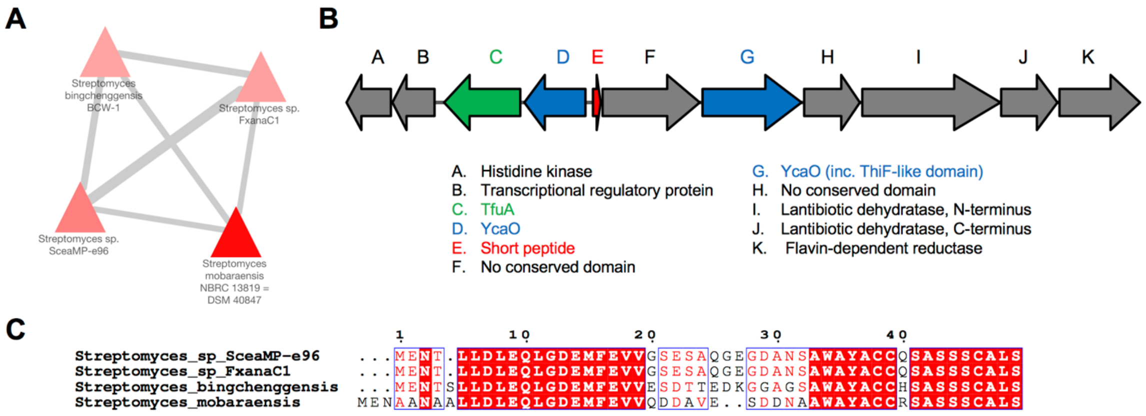

Figure S20. Network 28 overview. (A) Peptide network color-coded by Prodigal score. (B) Representative conserved BGC with PP, YcaO and TfuA genes highlighted. (C) Sequence alignment of all peptides present in network. Network and alignment color-coding is identical to Figure S8.

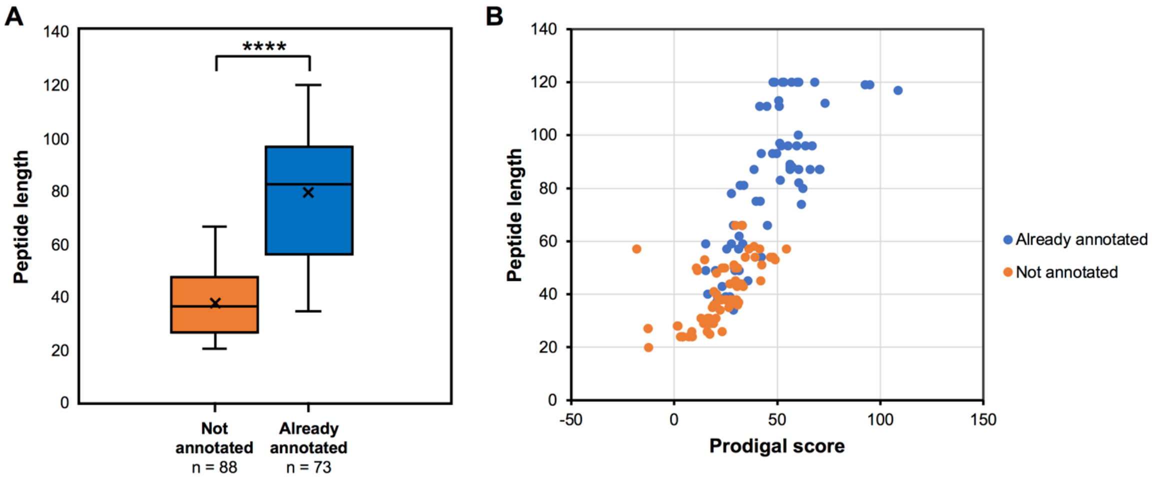

Figure S21. Analysis of whether peptides-coding ORFs detected by RiPPER were originally annotated. (A) Comparison of the size distributions of predicted precursor peptides from Networks 1, 2, 3, 5, 7, 9, 12, 15, 20, 21, 22, 24, 26, 27 and 28 that were either previously annotated or not annotated. **** = p-value <0.0001 (B) Plot of the peptide length versus the Prodigal score of these peptides.

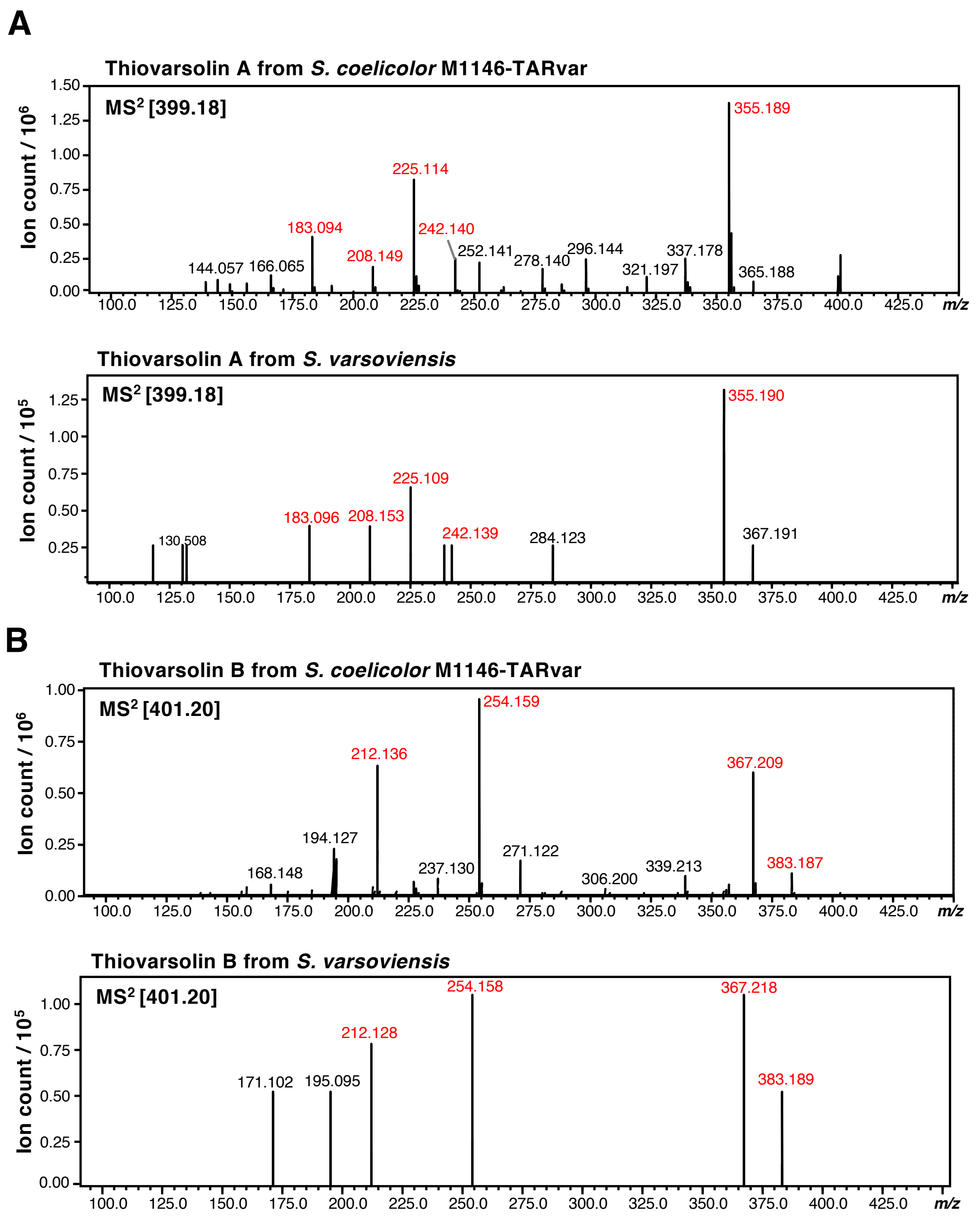

Figure S22. Comparsion of MS^2 data for thiovarsolins A (panel A) and B (panel B) produced by *S. coelicolor* M1146-TARvar and wild type *S. varsoviensis*. Fragments found in both strains are colored red.

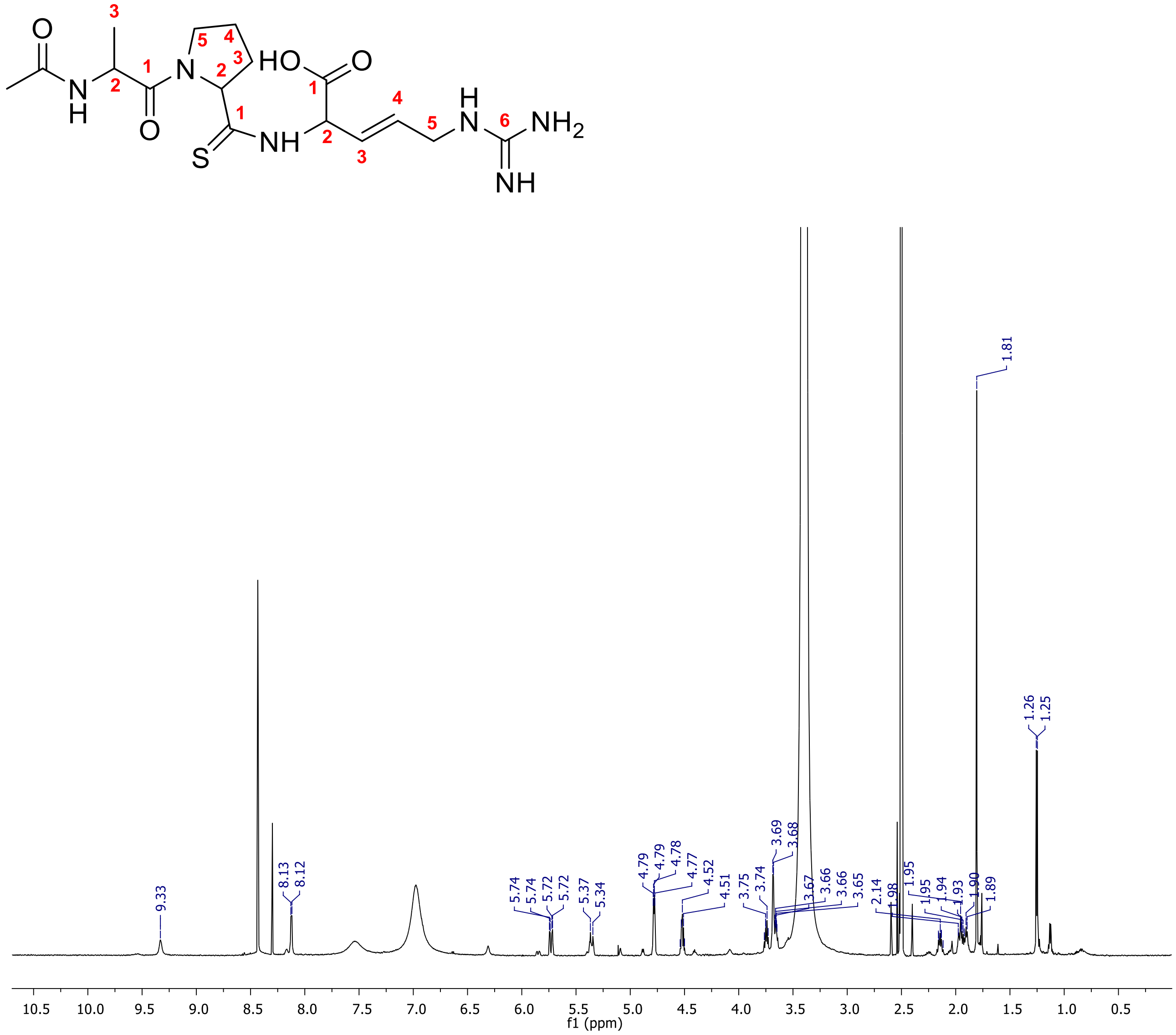

Figure S23. 700 MHz ^{1}H NMR spectrum of thiovarsolin A in DMSO-d_6. Carbon numbering used in Table S6 is also shown.

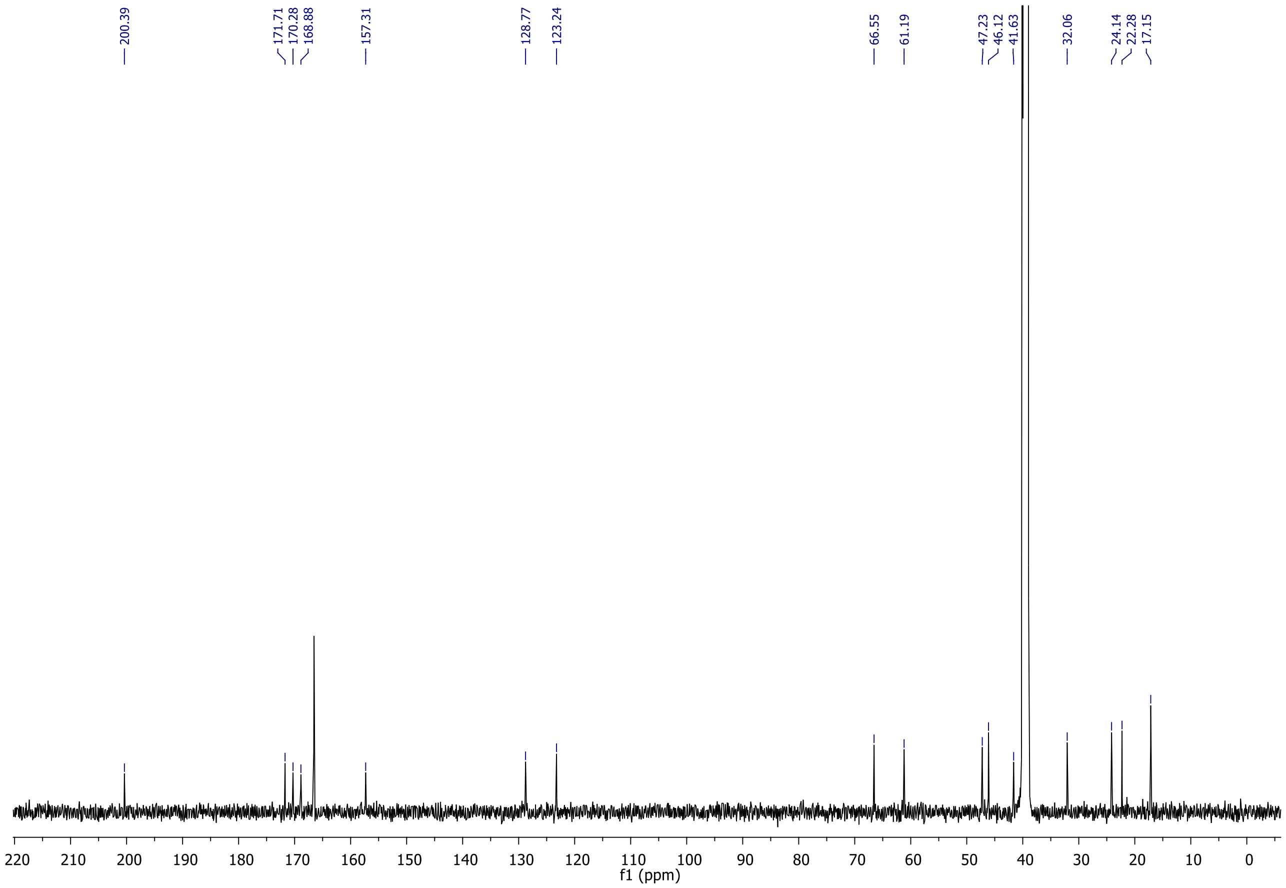

Figure S24. 175 MHz ^{13}C NMR spectrum of thiovarsolin A in DMSO-d_6.

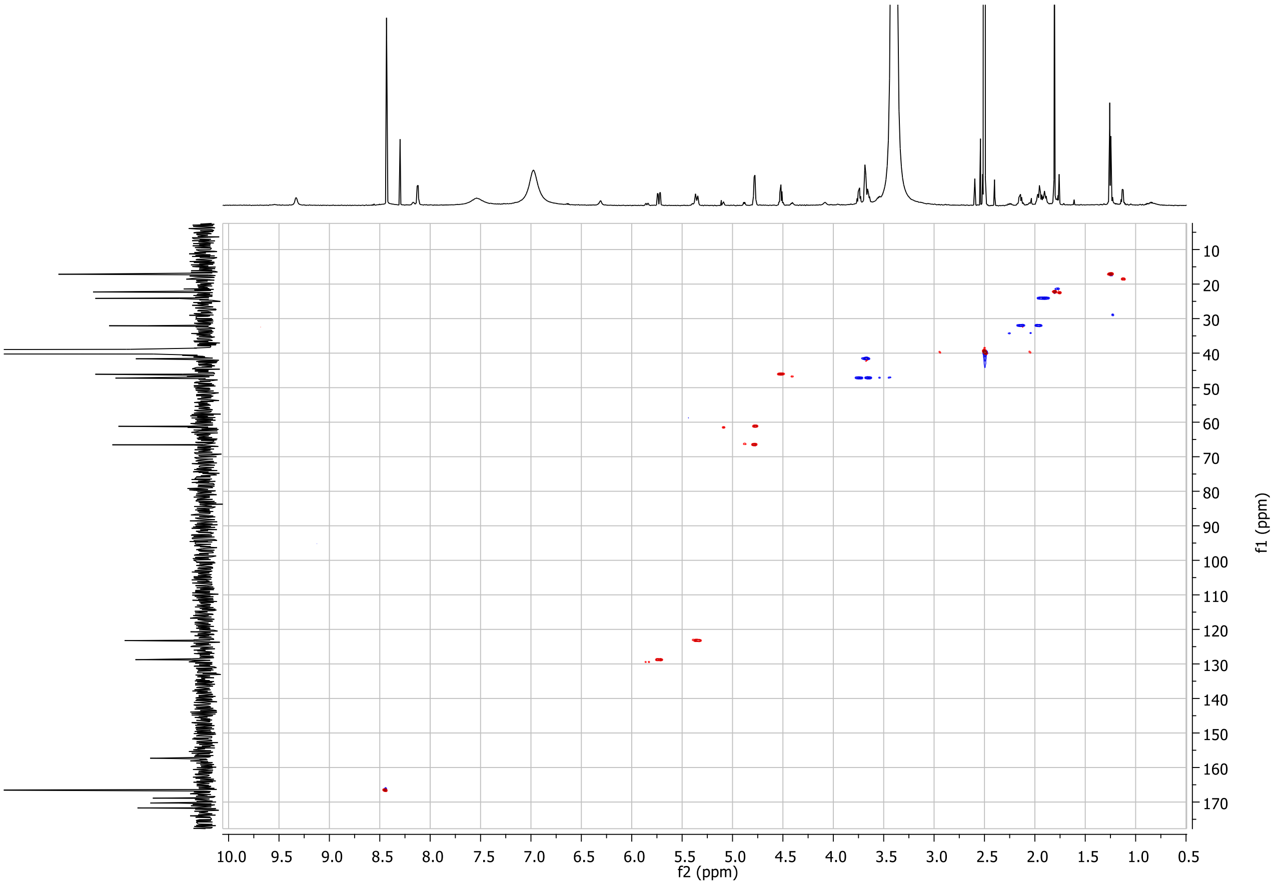

Figure S25. 700 MHz ^{1}H-^{13}C HSQC NMR spectrum of thiovarsolin A in DMSO-d_6.

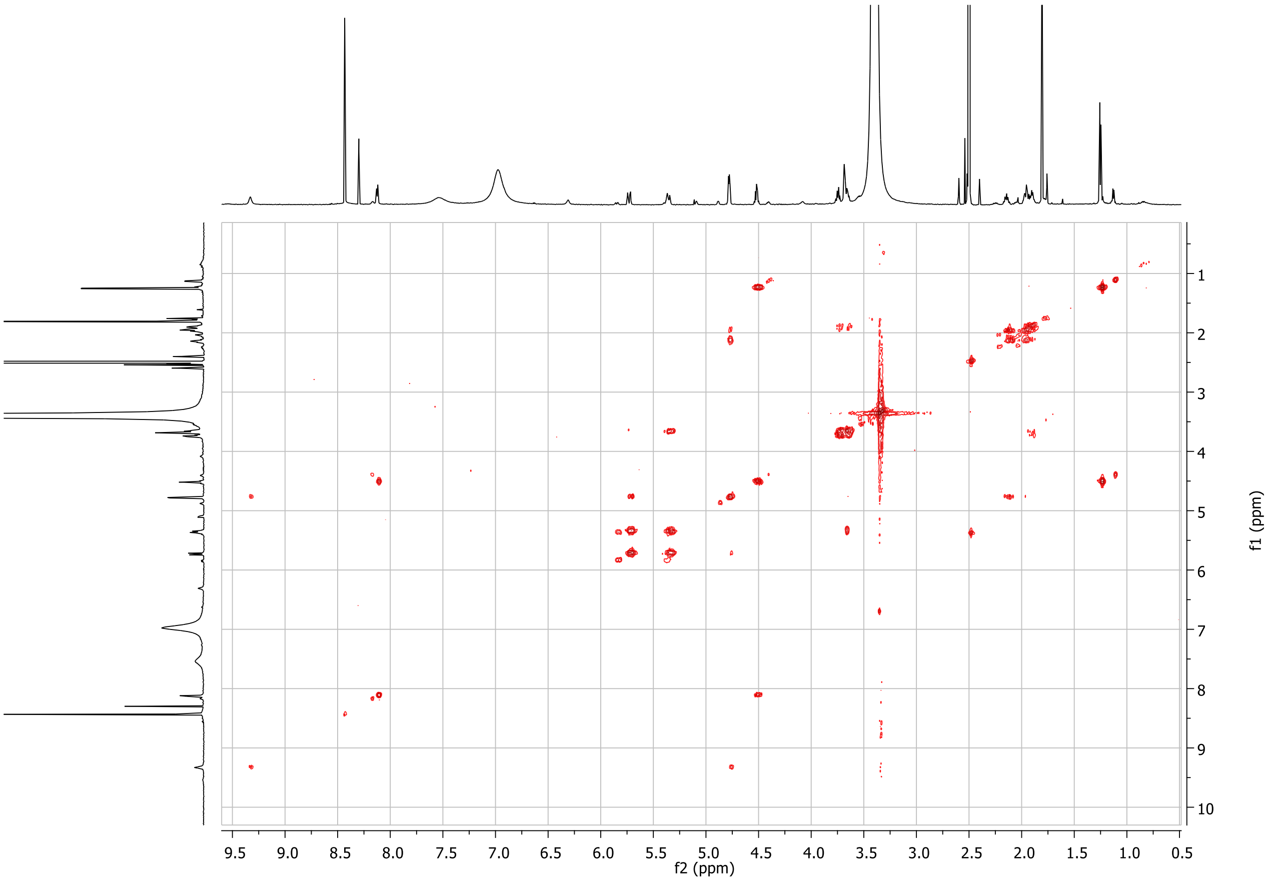

Figure S26. 700 MHz ^{1}H-^{1}H COSY NMR spectrum of thiovarsolin A in DMSO-d_6.

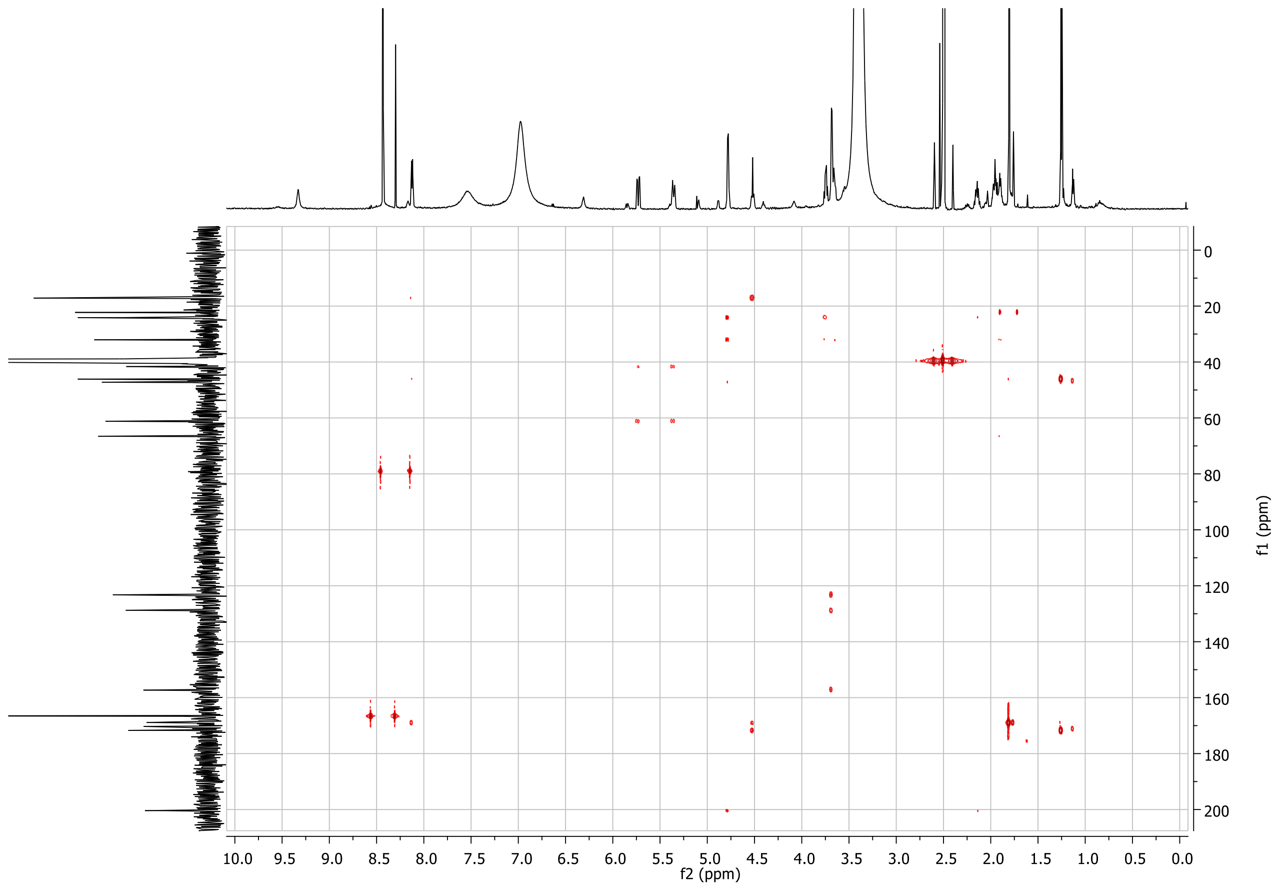

Figure S27. 700 MHz ^{1}H-^{13}C HMBC NMR spectrum of thiovarsolin A in DMSO-d_6.

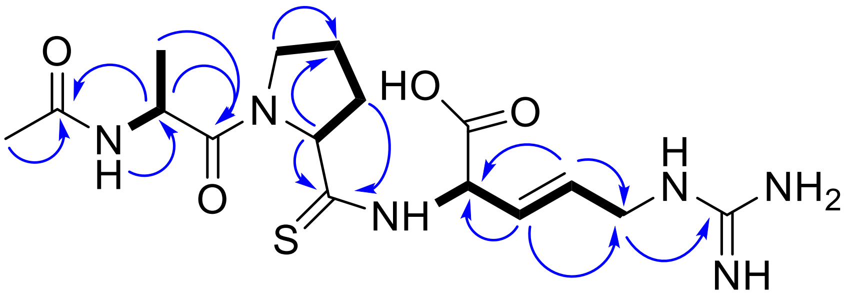

Figure S28. ^{1}H-^{1}H (COSY, bold lines) and ^{1}H-^{13}C (HMBC, blue arrows) correlations in thiovarsolin A.

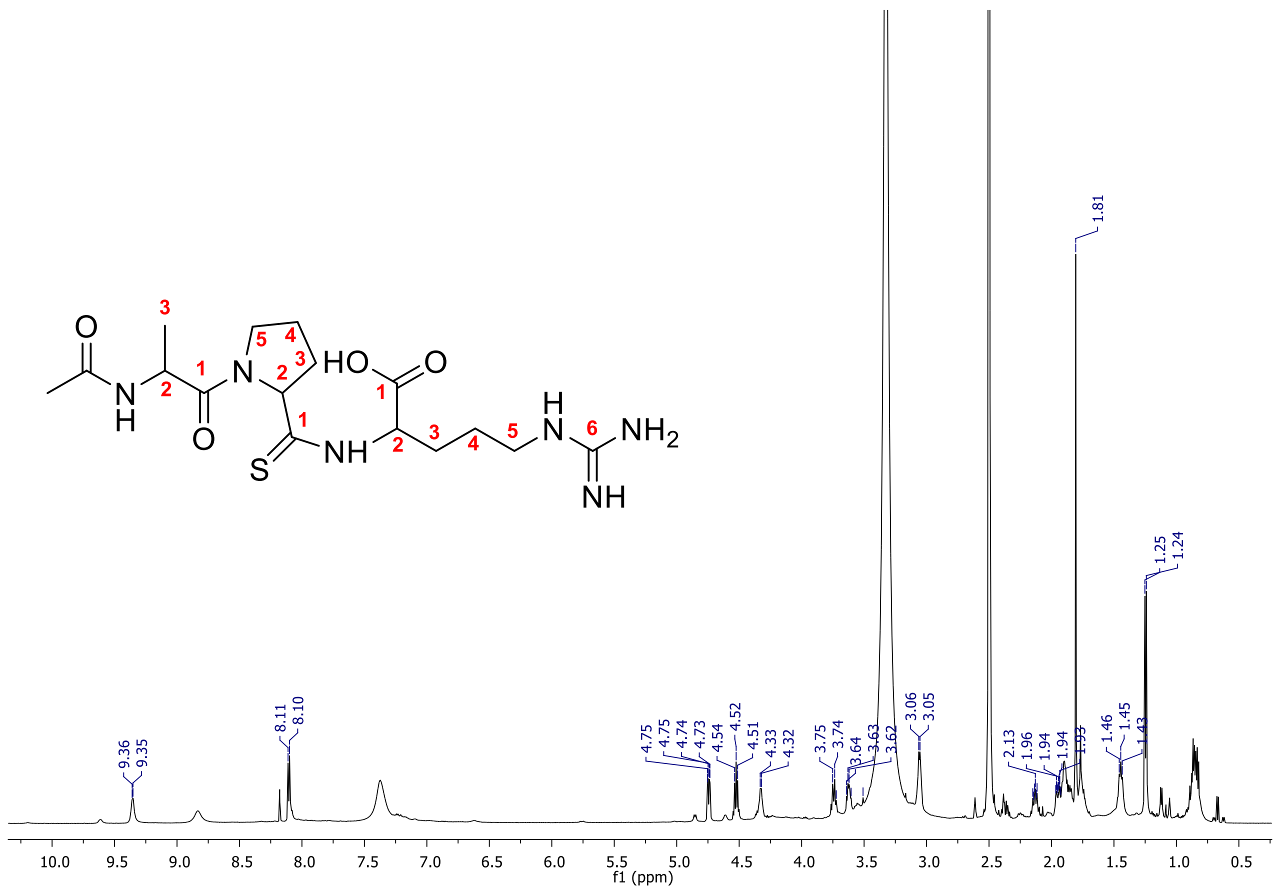

Figure S29. 600 MHz ^{1}H NMR spectrum of thiovarsolin B in DMSO-d_6.

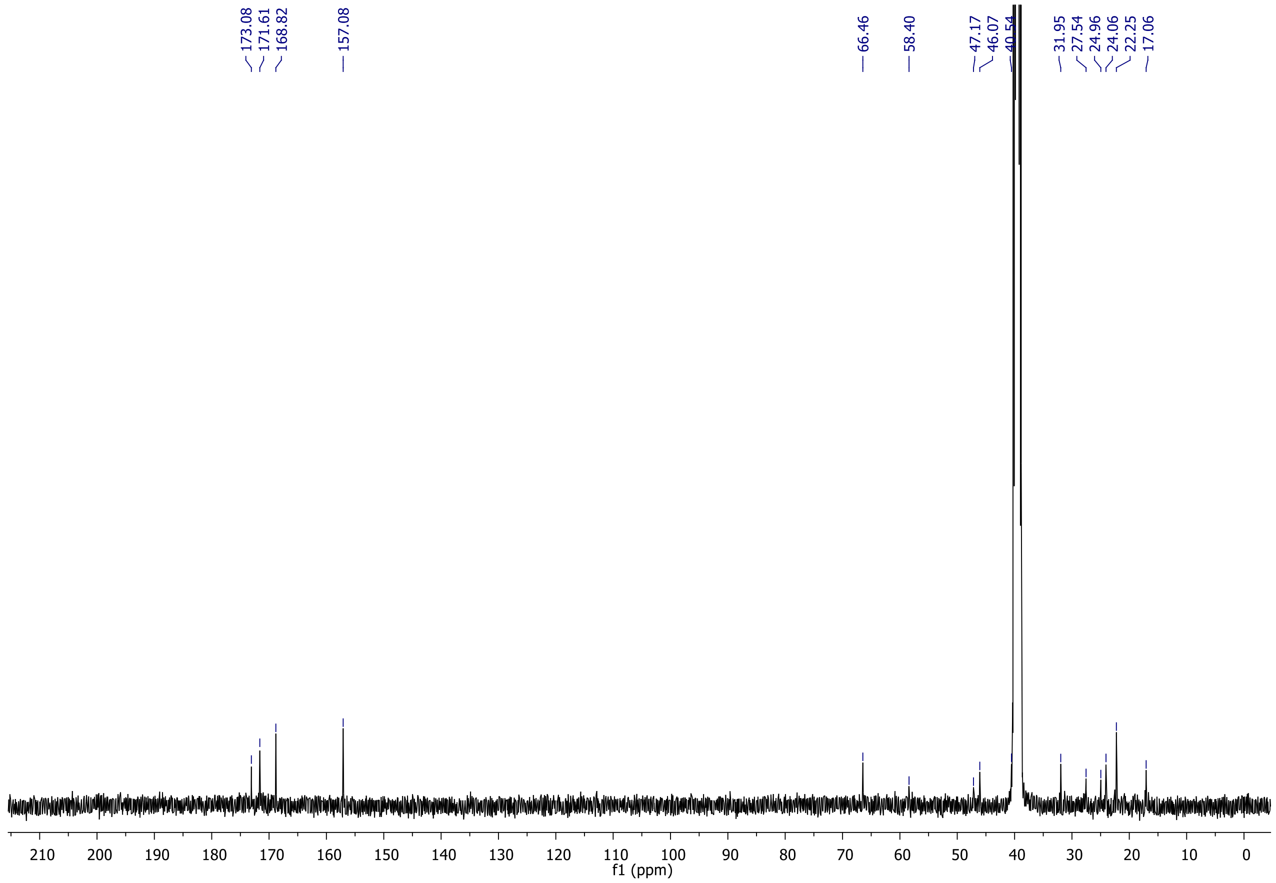

Figure S30. 100 MHz ^{13}C NMR spectrum of thiovarsolin B in DMSO-d_6. The thioamide carbonyl signal is not visible here but is clearly seen in the HMBC spectrum (Figure S32).

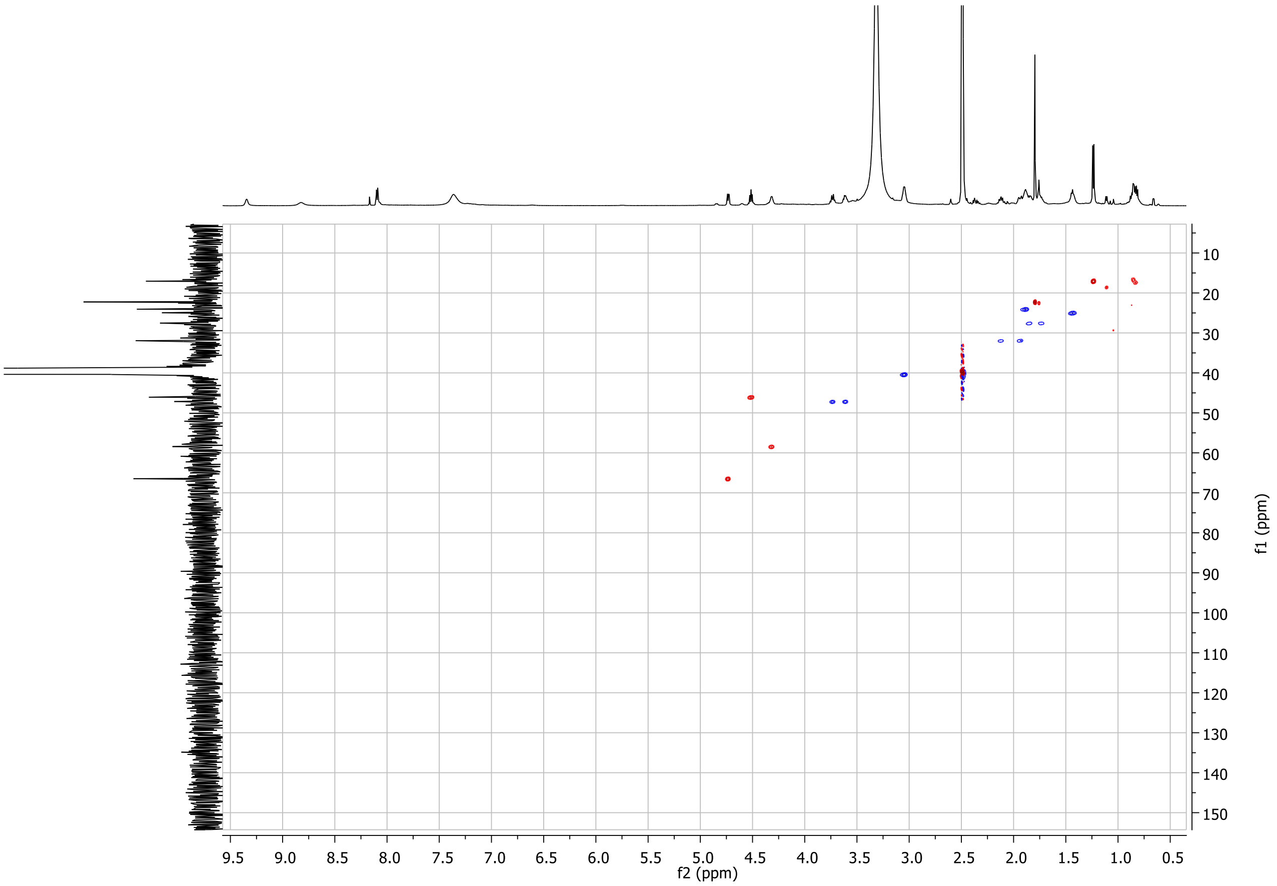

Figure S31. 600 MHz ^{1}H-^{13}C HSQC NMR spectrum of thiovarsolin B in DMSO-d_6.

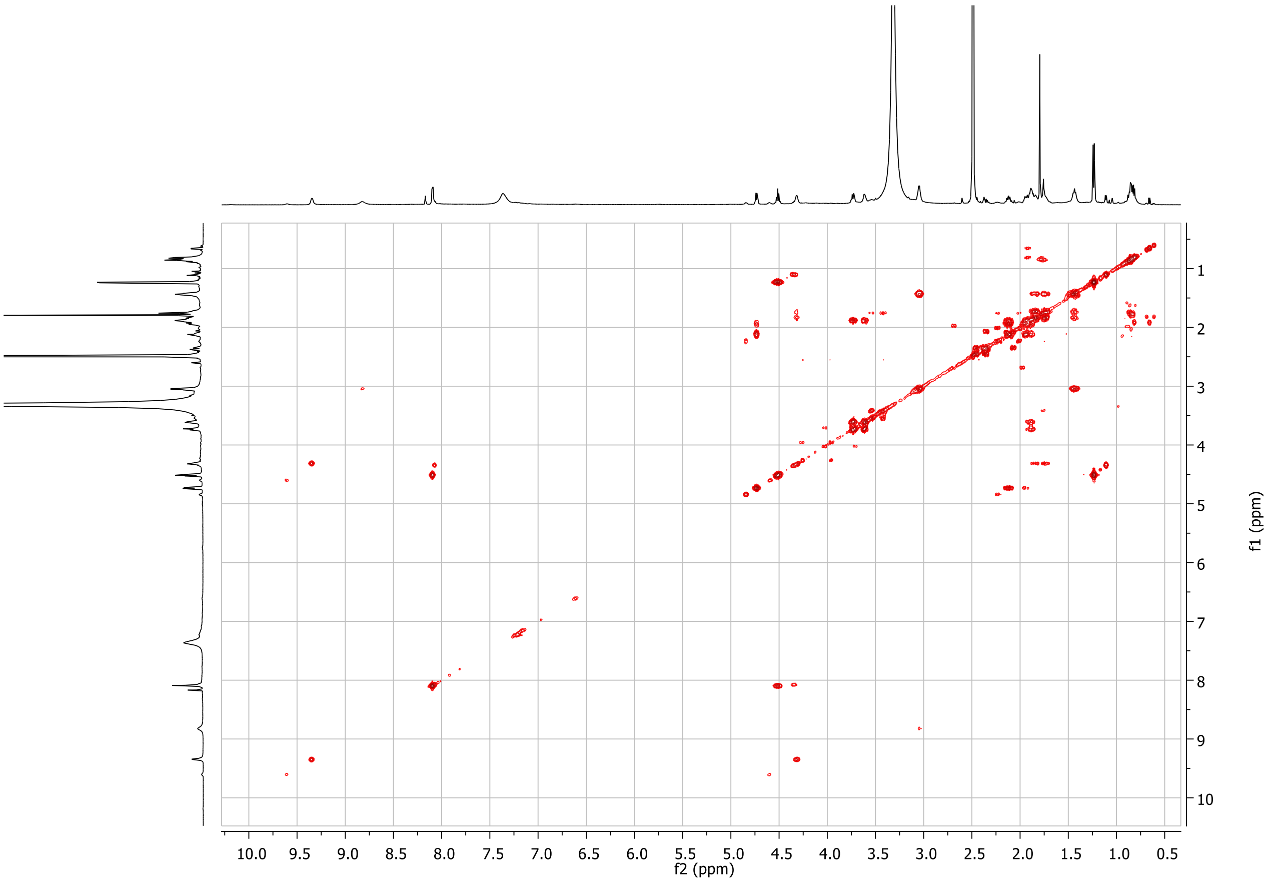

Figure S32. 600 MHz ^{1}H-^{1}H COSY NMR spectrum of thiovarsolin B in DMSO-d_6.

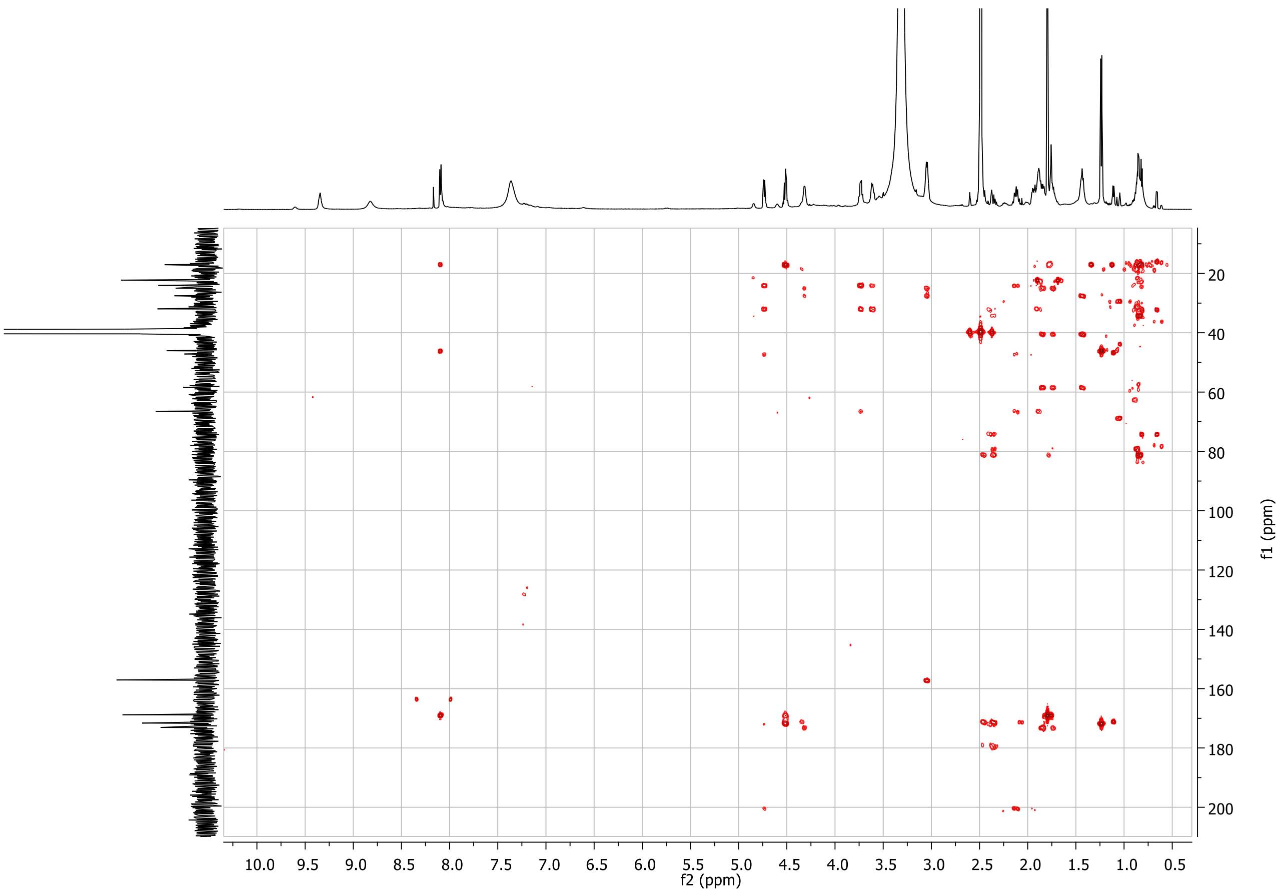

Figure S33. 600 MHz ^{1}H-^{13}C HMBC NMR spectrum of thiovarsolin B in DMSO-d_6.

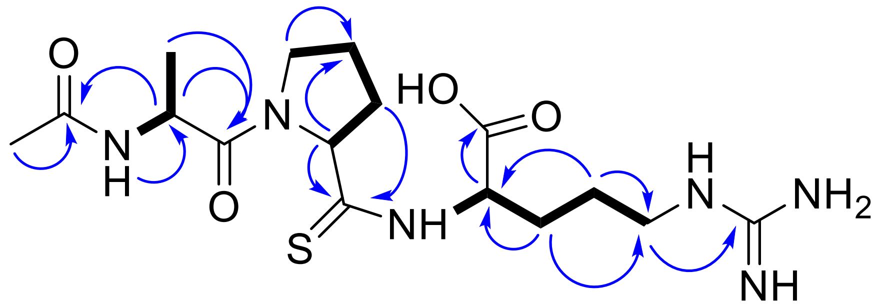

Figure S34. ^{1}H-^{1}H (COSY, bold lines) and ^{1}H-^{13}C (HMBC, blue arrows) correlations in thiovarsolin B.

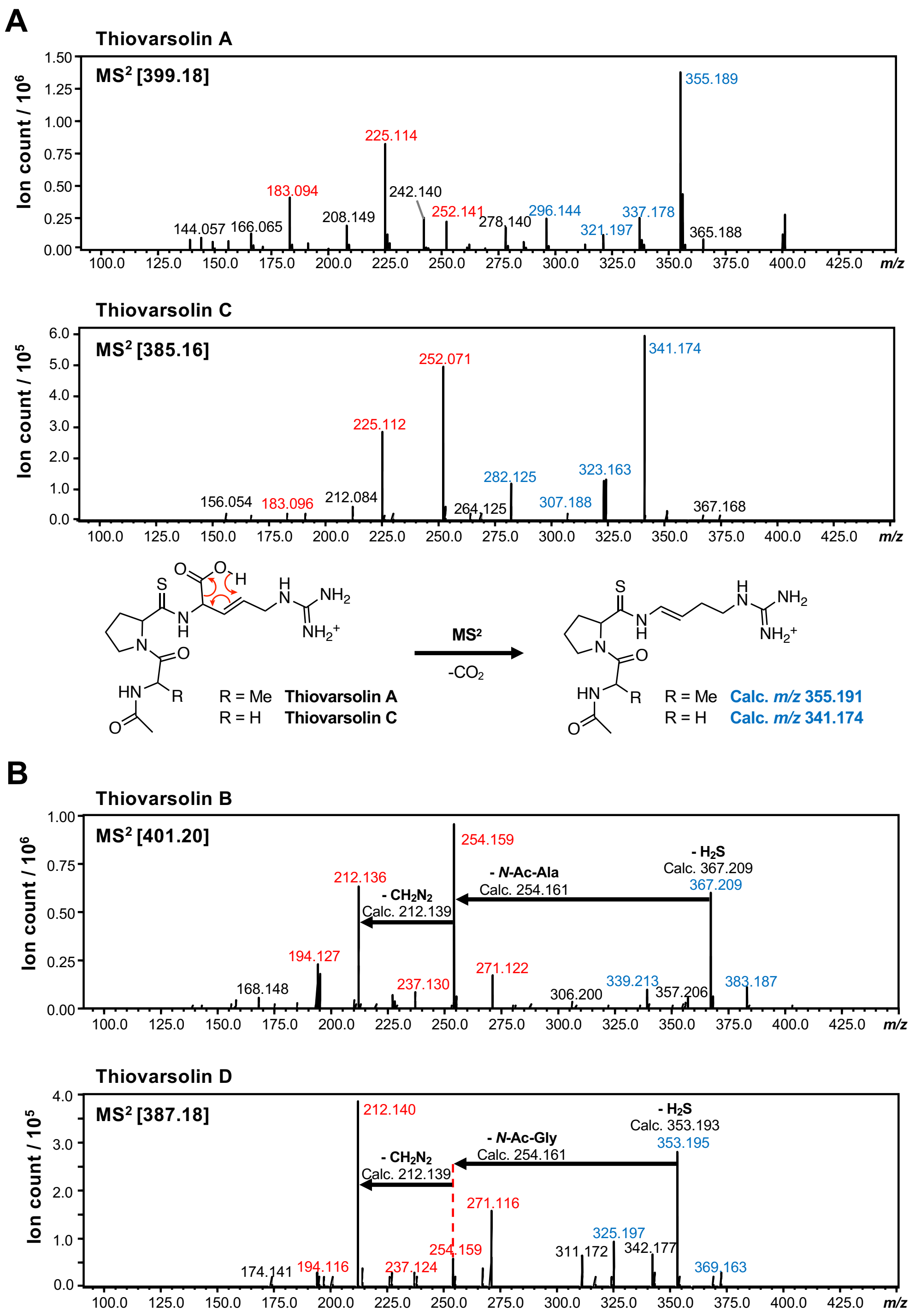

Figure S35. MS^2 analysis of thiovarsolins produced by *S. coelicolor* M1146-TARvar. (A) Comparison of MS^2 spectra of thiovarsolins A and C. The fragmentation data is consistent with a decarboxylation that may be assisted by the double bond on the arginine, as illustrated. (B) Comparison of MS^2 spectra of thiovarsolins B and D. In the absence of the Arg double bond, the loss of H_2S dominates, which is characteristic of thioamides.[22] Loss of the acetylated alanine or glycine provides common fragments (-CH_2N_2 corresponds to fragmentation on the Arg side chain and is equivalent to an arginine to ornithine fragmentation).

```
VarA      1 MRKDSDLRLDDLAHVNPEQLQGFLEERSTAAVHGATDAYFSGPRAQTVRG    50
            ||||||||||||||||||||||||||||||||||||||||||||||||||
VarA*     1 MRKDSDLRLDDLAHVNPEQLQGFLEERSTAAVHGATDAYFSGPRAQTVRG    50

VarA     51 ATDAHFSAPRAQTVRGATDAHFSAPRAQTVRGATDAHFSAPRAQTVRGAT   100
            ||||:||.|||||||||||..||.||||||||||||:||.||||||||||
VarA*    51 ATDAYFSGPRAQTVRGATDGTFSGPRAQTVRGATDAYFSGPRAQTVRGAT   100

VarA    101 DSYFSAPRAQNVR    113
            |||||.|||||||
VarA*   101 DSYFSGPRAQNVR    113
```

Figure S36. Sequence alignment of wild type VarA and the mutated VarA*, where each repeat has been modified to resemble the native GPR-containing repeat. The APR and GPR regions that the thiovarsolins derive from are highlighted in bold and are underlined, and the natural GPR-containing repeat is highlighted in red. Grey shading highlights a two-AA mutation that was a consequence of the approach required to construct such a repetitive gene.

SUPPLEMENTARY TABLES

Table S1. Summary of top 30 peptide networks identified for *tfuA*-containing BGCs in Actinobacteria.

Network	Consensus Pfam domain	Same strand	Notes	Likely precursor peptide?
**1**	None	Yes	*Mycobacteria*	**Possible**
**2**	None	Yes	*Streptomyces*	**Yes**
**3**	None	Mix	Mainly *Herbidospora*; some BGCs have two peptides in this network	**Possible**
**4**	None	Yes	*Streptomyces*; same BGCs as some network 2 peptides; not present in all BGCs with similar tailoring genes.	**No**
**5**	None	Yes	Mixed *Actinobacteria*; TLM network	**Yes**
**6**	None	No	*Mycobacteria*; same BGCs as some network 1 peptides; low peptide score	**No**
**7**	Nitrile hydratase	Yes	*Micromonospora*; Possible second related PP in each cluster not picked as over 120 AA	**Yes**
**8**	Nucleoporin C-terminal	No	*Mycobacteria*; same BGCs as some network 1 peptides	**No**
**9**	None	Yes	Mixed *Actinobacteria*	**Yes**
**10**	DUF732	No	*Mycobacteria*; same BGCs as some network 1 peptides	**Possible**
**11**	Ppant attachment site	Yes	Mixed *Actinobacteria*; same BGCs as some network 9 peptides	**No**
**12**	None	Yes	Mainly *Streptomyces*	**Yes**
**13**	PqqD	No	Mainly *Herbidospora*; same BGCs as some network 3 peptides	**No**
**14**	None	No	Mainly *Micromonospora*; very low peptide scores; same BGCs as some network 7 peptides	**No**
**15**	None	Yes	Mixed *Actinobacteria*	**Yes**
**16**	Helix-turn-helix	No	*Herbidospora*; same BGCs as some network 3 peptides	**No**
**17**	LuxR family	No	Mixed *Actinobacteria*; same BGCs as some network 3 peptides	**No**
**18**	ThiS family	Yes	*Streptomyces*; same BGCs as some network 9 peptides	**No**
**19**	DUF397	No	Mixed *Actinobacteria*	**No**
**20**	None	Yes	Mixed *Actinobacteria*; *Actinophytocola* entry likely an outlier (low score, limited homology)	**Yes**

**21**	None	Yes	Mixed *Actinobacteria*; Some BGCs have two peptides in this network	**Yes**
**22**	None	Yes	Mixed *Actinobacteria*; thiovarsolin network	**Yes**
**23**	DUF3017	Yes	*Mycobacteria*; same BGCs as some network 1 peptides	**Possible**
**24**	None	Yes	*Herbidospora*; three YcaO and one TfuA in each BGC	**Yes**
**25**	Trm112p	No	*Mycobacterium gordonae*; same BGCs as some network 1 peptides	**No**
**26**	None	No	Two *Amycolatopsis* species; one strain has three almost identical peptides; unusual gene position in relation to *ycaO-tfuA* genes	**Possible**
**27**	Ribosomally synthesized Frankia peptide	Yes	Mixed *Actinobacteria*	**Yes**
**28**	None	No	*Streptomyces*; peptides before series of RiPP biosynthesis genes on opposite strand (including an additional YcaO protein)	**Yes**
**29**	DUF2733	No	*Micromonospora*; very low peptide scores; same BGCs as some network 7 peptides	**No**
**30**	None	No	*Mycobacterium tuberculosis*; very low peptide scores; same BGCs as some network 1 peptides	**No**

Table S2. ORFs and associated predicted protein functions in the pTARvar insert.

ORF	Accession	Length (bp)	Predicted function	Conserved domain	Mutant (PCR targeting primers)	Effect on thiovarsolin production
1	WP_030877102.1	2097	Peptidase	PF02897	ΔLeft_arm1 (RD16 and RD17)	No
2	WP_030877105.1	1101	AraC regulator / Cupin 6	PF12833/ PF12852	ΔLeft_arm1	No
3	WP_078644544.1	1278	Major Facilitator Superfamily	PF07690	ΔLeft_arm1	No
4	WP_078644578.1	1194	Methyltransferase	PF01135	ΔLeft_arm1	No
5	WP_030877113.1	567	Nucleoside deaminase	PF00383	ΔLeft_arm1	No
6	WP_030877115.1	1962	NRPS (A/T domains)	PF00501/ PF00550	ΔLeft_arm2 (RD18 and RD19)	No
7	WP_030877117.1	1407	Halogenase	PF04820	ΔLeft_arm2	No
8	WP_030877120.1	972	Thioesterase	PF00975	ΔLeft_arm2	No
9 (*varO*)	WP_030877123.1	690	Heme oxygenase	PF14518	Δ*varO* (RD14 and RD15)	**Yes**
10 (*varS*)	WP_030877126.1	1134	Amidinotransferase[a]	Not assigned	Δ*amdT* (RD12 and RD13)	No
11 (*varA*)	WP_030877127.1	342	Precursor Peptide	Not assigned	Δ*varA* (RD1 and RD3) Δ*varA*core (RD2 and RD3)	**Yes**
12 (*varY*)	WP_030877129.1	1347	YcaO-domain protein	PF02624	Δ*varY* (RD4 and RD5)	**Yes**
13 (*varT*)	WP_063763981.1	816	TfuA-like protein	PF07812	Δ*varT* (RD6 and RD7)	**Yes**
14 (*varP*)	WP_063763980.1	1452	Major Facilitator Superfamily	PF07690	Δ*mfs* (RD8 and RD9)	No
15 (*varL*)	WP_030877136.1	1362	ATP-grasp ligase[a]	Not assigned	Δ*atp_gl*	No

					(RD10 and RD11)	
16	WP_030877138.1	999	Metallo-dependent hydrolase	PF04909	ΔRight_arm (RD20 and RD21)	No
17	WP_030877139.1	753	Short chain Acyl-CoA dehydrogenase	PF13561	ΔRight_arm	No
18	WP_030877141.1	318	Hypothetical protein	Not assigned	ΔRight_arm	No
19	WP_030877143.1	1290	Membrane protein	PF10011	ΔRight_arm	No
20	WP_048832106.1	1245	Acyl-CoA transferase	PF02515	ΔRight_arm	No
21	WP_078644541.1	1653	Major Facilitator Superfamily	PF07690	ΔRight_arm	No
22	WP_030877149.1	687	GntR family regulator	PF00392	ΔRight_arm	No
23	WP_030877152.1	849	Formate dehydrogenase associated protein	PF02634	ΔRight_arm	No
24	WP_030877153.1	474	MarR family regulator	PF12802	ΔRight_arm	No
25	WP_030877155.1	933	NADPH-quinone reductase	PF00107	ΔRight_arm	No

[a] Functions predicted by homology to proteins in pheganomycin biosynthesis (10).

Table S3. Oligonucleotides used in this study.

Name[a]	Sequence[b] (5'→3')
TARvar_1	**TTTGACGCCTCCCATGGTATAAATAGTGGCTCGAGGGGTCGCGGCCTTCTCCGTACCCGCAGCGCTCATCGCCA**CCTCCGCGGGAGTTTAAACCAGGCGATGGC
TARvar_2	**AGCAGCACGTTCCTTATATGTAGCTTTCGACATATGCGAGCAGCGCTCCCGAGGCCAGGGTCAAGGCCCGCCGCAGCCATCGCCT**GGTTTAAACTCCCGCGGAGG
CAP03_check-fw	CCGCCTTTTCCTCAATCGCTCTTC
CAP03_check-rv	GGACATATCCACGCCCTCCTACATCG
TAR_check-fw	GATACAGGATCCGTCATCCCGAGATCCAACGAC
TAR_check-rv	GATACAGAATTCCGATCACCCGTACCACGTTGACCG
RD1 (RD_*varA*core-fw)	**CAAGGCTTCCTGGAGGAGCGCTCCACCGCCGCCGTCCAC**ATTCCGGGGATCCGTCGACC
RD2 (RD_*varA*-fw)	**CGGCCTCCAAACCCGTAAGGAAAGGAAAAGGCCCTCATG**GCAGCTCACGGTAACTGATGC
RD3 (RD_*varA*-rv)	**GTGGTCTTTCCGTGGTCTTCCGTGGTCTTCGGGGGCTCA**TGTAGGCTGGAGCTGCTTC
RD4 (RD_*varY*-fw)	**CACAGACACGGAGATGGAGACGGAGACAGCGTGAAGATG**GCAGCTCACGGTAACTGATGC
RD5 (RD_*varY*-rv)	**GGCTGAGGGGGACTGGGCTGAGGCGAGAGACGCGTGTCA**TGTAGGCTGGAGCTGCTTC
RD6 RD_varT-fw:	**GCCTCCGGGCCTGCCTAGCATCTGGGAGCGTTCTCCATG**GCAGCTCACGGTAACTGATGC
RD7 RD_varT-rv	**CCGTGGCCGTGGCCCTGCCGCTGTCCGGAGTCTTCTCC**TGTAGGCTGGAGCTGCTTC
RD8 RD_msf-fw	**GCACACGCCGCCGGGCCGCTTCGAAAGGGCCCGTTCTTG**GCAGCTCACGGTAACTGATGC
RD9 RD_msf-rv	**GGACTCGCGATCGACCACCGCGCCGAAGACGGGCTTCA**TGTAGGCTGGAGCTGCTTC
RD10 RD_*atp-gl*-fw	**ATCACCCGCGCCCCCACCCCGAGAAGGGAAGCCACGATG**GCAGCTCACGGTAACTGATGC
RD11 RD_*atp-gl*-rv	**GGCGGCGTGGGCCCCAGCCCCACTCGGCAGGGCGGTCA**TGTAGGCTGGAGCTGCTTC
RD12 RD_*amt*_fw	**CCCGGTCCTTTCGAAGCCCCAGAGGAGAGCCCATCGATG**GCAGCTCACGGTAACTGATGC
RD13 (RD_*amt*-rv)	**CTATGGCGGCCGCGCCCGTGGCCCGCGCCCGGCGGTCA**TGTAGGCTGGAGCTGCTTC
RD14 (RD_*varO*-fw)	**GACGAACAACGACGGAAAGCGAGTGGTGGGCGAGCGATG**GCAGCTCACGGTAACTGATGC

RD15 (RD_*varO*-rv)	**CACGGACATGAACATGAACAAGGACAAGGACATGTTCA**TGTAGGCTGGAGCTGCTTC
RD16 (RD_left_arm1-fw)	**TGAGGAGCACCGCACGCGGGCGGTCCCGTTCGGCGTCTA**GCAGCTCACGGTAACTGATGC
RD17 (RD_left_arm1-rv)	**GAGCGTCCCCGGGCGCCGTCCCACAGGCCCGTCGTCA**TGTAGGCTGGAGCTGCTTC
RD18 (RD_left_arm2-fw)	**CGGCGGCCGGCCCCGACGCGGCCTCGGCGGGCCGCGGTG**GCAGCTCACGGTAACTGATGC
RD19 (RD_left_arm2-rv)	**TAGACGCCGAACGGGACCGCCCGCGTGCGGTGCTCCTCA**TGTAGGCTGGAGCTGCTTC
RD20 (RD_right_arm-fw)	**CCGCCCACCGGCCGCCGGCCGCCGCCCAGGTGATCGTCA**GCAGCTCACGGTAACTGATGC
RD21 (RD_right_arm-rv)	**GCACCCACAAGAACCGCACGTCACCTGACTGGGGATCA**TGTAGGCTGGAGCTGCTTC
CK1 (*varA*_check-fw)	CGCACAACTCGGCAGAGGCGG
CK2 (*varA*_check-rv)	CGGCGGACAGGGACTCGAC
CK3 (*varY*_check-fw)	GCCAAGTCGAGTCCCTGTCCG
CK4 (*varY*_check-rv)	GGTGAGGCGAGGGGACTGAGAGAG
CK5 (*varT*_check-fw)	CCCCTCGCCTCACCGTCCTCTTTC
CK6 (*varT*_check-rv)	GGAGACCAGCAGGACGACGC
CK7 (*msf*_check-fw)	GCCACGGATTCCGACACGGTC
CK8 (*msf*_check-rv)	GCGGGTGATTCGGCTGATTTGG
CK9 (*atp-gl*_check-fw)	CCAAATCAGCCGAATCACCCGC
CK10 (*atp-gl*_check-rv)	CGGTGGGCGGACGGTCG
CK11 (*amT*_check-fw)	CGTTGTTCGTCGCTTTCCCTATGG
CK12 (*amT*_check-rv)	CCGTCCCGTCCCCGATGC
CK13 *varO*_check-fw)	CCATAGGGAAAGCGACGAACAACG
CK14 (*varO*_check-rv)	CATGGACATGAACATGGACATGGC
CK15 (left_arm1_check-fw)	GGGAAGCCTACACCGTGCAGTG
CK16 (left_arm1_check-rv)	CGCTGTTCGCCACCGTGCTCG
CK17 (left_arm2_check-fw)	GCCCGAGAAGCACCCCTACTAGAC
CK18 (left_arm2_check-rv)	CCTTGTTCATGTTCATGTCCGTGGC
CK19 (right_arm_check-fw)	CGACCGTCCGCCCACCG
CK20 (right_arm_check-fw)	GAACCGCACGTCACCTGACTGG

CP1 (*varA*_comp-fw)	GATACACATATGAGGAAAGACTCGGATCTCAGGC
CP2 (*varA*_comp-rv)	GATACAAAGCTTCTGTGCCTGTCTGTCTTCGTATTTCTCTG
CP3 (*varA*p_comp-fw)	GATACACATATGGGGCTCTCCTCTGGGGCTTCG
CP4 (*varA*_comp-rv2)	GATACAAAGCTTCCCGCATCTTCACGCTGTCTCC
CP5 (*varY*_comp-fw)	GATACACATATGCGGGCTCACGCCCCC
CP6 (*varY*_comp-rv)	GATACAAAGCTTGGAGAACGCTCCCAGATGCTAGG
CP7 (*varT*_comp-fw)	GATACACATATGGGACCCGGTGGTCTTCCTCG
CP8 (*varT*_comp-rv)	GATACAAAGCTTCGAGCCCGAGCATGGGTACG
CP9 (*varO*_comp-fw)	GATACACATATGACGGAGAGTCTGGACCGGG
CP10 (*varO*_comp-rv)	GATACAAAGCTTCGTGAGGTCGGTCAGCAGCG
AG1	GTGGACGGCGGCGGTGG
AG2	CCGCGCACCGTCTGGGCCCGCGGGCCCGAGAAGTACGCGTCCGTCGCG CCGTGGACGGCGGCGGTGG
AG3	GATACATCTAGAGGTACCGTCGGTCGCCCCCCGGACGGTCTGCGCCCGC GGCCCGCTGAAGTAGGCGTCGGTCGCCCCGCGCACCGTCTGGGC
AG4	GTCCGCTGAGCCCCCGAAGACC
AG5	GGCGCCACGGACTCCTACTTCTCCGGACCGCGGGCCCAGAACGTCCGCT GAGCCCCCGAAGACC
AG6	GATACAGGTACCTTCAGCGGGCCGCGGGCGCAGACCGTCCGGGGGGCG ACCGACGCCTACTTCAGCGGGCCGCGGGCGCAGACCGTCCGGGGCGCC ACGGACTCCTACTTCTCC

[a] Primer naming code: RD = primers for PCR targeting; CK = primers for mutant checking; CP = primers for the complementation of mutants; AG = primers for alanine → glycine substitution in *varA*.

[b] Bold letters indicate homologous recombination regions. Underlined letters represent restriction sites.

Table S4. Vectors and constructs used in this study.

Vector / construct	Relevant features	Use	Source or reference
pCAP03	kan^R, *oriT*, ΦC31 int-attP	Capture of the thiovarsolin BGC	11
pTARvar	kan^R, *oriT*, ΦC31 int-attP, 31.7 kb insert from *S. varsoviensis*	Heterologous pathway expression	This work
pTARvar Δ*varA*_clean	kan^R, *oriT*, ΦC31 int-attP, in-frame deletion of the *varA* core peptide	Functional analysis of *varA*	This work
pTARvar Δ*varA*	Same as pTARvar, insertional deletion of *varA*	Functional analysis of *varA*	This work
pTARvar Δ*varY*	Same as pTARvar, insertional deletion of *varY*	Functional analysis of *varY*	This work
pTARvar Δ*varT*	Same as pTARvar, insertional deletion of *varT*	Functional analysis of *varT*	This work
pTARvar Δ*varO*	Same as pTARvar, insertional deletion of *varO*	Functional analysis of *varO*	This work
pTARvar Δ*amT*	Same as pTARvar, insertional deletion of *amT*	Functional analysis of *amT*	This work
pTARvar Δ*atp_gl*	Same as pTARvar, insertional deletion of *varA*	Functional analysis of *atp_gl*	This work
pTARvar Δ*mfs*	Same as pTARvar, insertional deletion of *mfs*	Functional analysis of *mfs*	This work
pTARvar Δleft_arm1	Same as pTARvar, insertional deletion of 5 genes in the left arm	Functional analysis of multiple genes	This work
pTARvar Δleft_arm2	Same as pTARvar, insertional deletion of 3 genes in the left arm	Functional analysis of multiple genes	This work
pTARvar Δright_arm	Same as pTARvar, insertional deletion of 10 genes in the left arm	Functional analysis of multiple genes	This work
pIJ773	amp^R, apra^R and *oriT* flanked by FRT sites	Template for PCR – targeting cassette	12

pIJ773 Δ*oriT*	amp^R, apra^R flanked by FRT sites	Template for PCR – targeting cassette	John Innes Centre Collection
pIJ10257	hyg^R, oriT, ϕBT1 int-attB, ermEp*	Constitutive expression in *Streptomyces*	13
pIJ10257_*varA*	Same as pIJ10257, carrying varA	*ΔvarA* complementation	This work
pIJ10257_*varA*p	Same as pIJ10257, carrying *varA* and its native promoter	ΔvarA complementation (under *var A* native promoter)	This work
pIJ10257_*varY*	Same as pIJ10257, carrying *varY*	*ΔvarY* complementation	This work
pIJ10257_*varT*	Same as pIJ10257, carrying *varT*	*ΔvarT* complementation	This work
pIJ10257_*varO*	Same as pIJ10257, carrying *varO*	*ΔvarO* complementation	This work
pIJ10257_*varA*p*YT*	Same as pIJ10257, carrying am operon comprising *varA*, *varY* and *varT*.	Expression of the minimal thiovarsolins BGCs (under the *varA* native promoter)	This work
pGP9	apra^R, oriT, ϕBT1 int-attB, Act-ORFIV / P_{actI} activator / promoter	Constitutive expression in *Streptomyces*	1
pGP9_*varA**p	pGP9-based, carrying *varA* with a point mutation.	*ΔvarA* complementation with a mutant peptide	This work

Table S5. Accurate mass data for thiovarsolins A-D.

Compound	$[M+H]^+$ formula	Expected *m/z*	Observed *m/z*	Mass error (ppm)
Thiovarsolin A	$C_{16}H_{27}N_6O_4S^+$	399.1809	399.1818	+2.25
Thiovarsolin B	$C_{16}H_{29}N_6O_4S^+$	401.1966	401.1968	+0.50
Thiovarsolin C	$C_{15}H_{25}N_6O_4S^+$	385.1653	385.1652	-0.26
Thiovarsolin D	$C_{15}H_{27}N_6O_4S^+$	387.1809	387.1808	-0.26

Table S6. ^{1}H and ^{13}C NMR data for thiovarsolins A and B in DMSO-d_6.

	Thiovarsolin A		Thiovarsolin B	
**Residue**[a]	**δ_C**	**δ_H, mult., *J* in Hz**	**δ_C**	**δ_H, mult., *J* in Hz**
**Ala**				
1	171.7, C	-	171.6, C	-
2	46.1, CH	4.52, dd, 7.2, 7.0	46.1, CH	4.52, dq, 7.0, 7.0
3	17.2, CH_3	1.25, d, 7.0	17.1, CH_3	1.25, d, 7.0
NH	-	8.12, d, 7.2	-	8.11, d, 7.0
CO	168.9, C	-	168.8, C	-
CH_3	22.3, CH_3	1.81, s	22.3, CH_3	1.81, s
**Pro**				
1	200.4, C	-	200.2, C[b]	-
2	66.6, CH	4.78, dd, 7.9, 4.5	66.5, CH	4.74, dd, 8.6, 3.0
3	32.1, CH_2	1.95, m<br>2.14, m	32.0, CH_2	1.95, ddd, 11.5, 8.6<br>2.13, m
4	24.1, CH_2	1.92, m	24.1, CH_2	1.87, m
5	47.2, CH_2	3.66, m<br>3.75, dd, 14.6, 7.4	47.2, CH_2	3.62, dd, 14.0, 8.0<br>3.74, dd, 14.0, 8.6
**Arg'**				
1	170.3, C	-	173.1, C	-
2	61.2, CH	4.78, dd, 7.9, 4.5	58.4, CH	4.33, dd, 6.4, 5.7
3	128.8, CH	5.73, dd, 15.6, 7.9	27.5, CH_2	1.74, m<br>1.84, m
4	123.4, CH	5.36, dd, 15.6, 4.5	25.0, CH_2	1.42, m
5	41.6, CH_2	3.68, d, 4.5	40.5, CH_2	3.08, m
6	157.3, C	-	157.1, C	-
NH	-	9.33, br s		9.35, d, 6.4

[a] Carbon numbering is shown in Figures S23 and S29.

[b] Assigned based on HMBC correlations (Figure S33).

REFERENCES

1. Kuščer,E., Coates,N., Challis,I., Gregory,M., Wilkinson,B., Sheridan,R. and Petkovic,H. (2007) Roles of *rapH* and *rapG* in positive regulation of rapamycin biosynthesis in *Streptomyces hygroscopicus*. *J. Bacteriol.*, **189**, 4756–4763.
2. Carver,T., Harris,S.R., Berriman,M., Parkhill,J. and McQuillan,J.A. (2012) Artemis: an integrated platform for visualization and analysis of high-throughput sequence-based experimental data. *Bioinformatics*, **28**, 464–469.
3. Medema,M.H., Takano,E. and Breitling,R. (2013) Detecting sequence homology at the gene cluster level with MultiGeneBlast. *Mol. Biol. Evol.*, **30**, 1218–1223.
4. Tietz,J.I., Schwalen,C.J., Patel,P.S., Maxson,T., Blair,P.M., Tai,H.-C., Zakai,U.I. and Mitchell,D.A. (2017) A new genome-mining tool redefines the lasso peptide biosynthetic landscape. *Nat. Chem. Biol.*, **13**, 470–478.
5. Ahmed,M.N., Reyna-González,E., Schmid,B., Wiebach,V., Süssmuth,R.D., Dittmann,E. and Fewer,D.P. (2017) Phylogenomic Analysis of the Microviridin Biosynthetic Pathway Coupled with Targeted Chemo-Enzymatic Synthesis Yields Potent Protease Inhibitors. *ACS Chem. Biol.*, **12**, 1538–1546.
6. Schwalen,C.J., Hudson,G.A., Kille,B. and Mitchell,D.A. (2018) Bioinformatic Expansion and Discovery of Thiopeptide Antibiotics. *J. Am. Chem. Soc.*, **140**, 9494–9501.
7. Halary,S., McInerney,J.O., Lopez,P. and Bapteste,E. (2013) EGN: a wizard for construction of gene and genome similarity networks. *BMC Evol. Biol.*, **13**, 146.
8. Frattaruolo,L., Lacret,R., Cappello,A.R. and Truman,A.W. (2017) A Genomics-Based Approach Identifies a Thioviridamide-Like Compound with Selective Anticancer Activity. *ACS Chem. Biol.*, **12**, 2815–2822.
9. Robert,X. and Gouet,P. (2014) Deciphering key features in protein structures with the new ENDscript server. *Nucleic Acids Res.*, **42**, W320–W324.
10. Noike,M., Matsui,T., Ooya,K., Sasaki,I., Ohtaki,S., Hamano,Y., Maruyama,C., Ishikawa,J., Satoh,Y., Ito,H., *et al.* (2015) A peptide ligase and the ribosome cooperate to synthesize the peptide pheganomycin. *Nat. Chem. Biol.*, **11**, 71–76.
11. Tang,X., Li,J., Millán-Aguiñaga,N., Zhang,J.J., O'Neill,E.C., Ugalde,J.A., Jensen,P.R., Mantovani,S.M. and Moore,B.S. (2015) Identification of Thiotetronic Acid Antibiotic Biosynthetic Pathways by Target-directed Genome Mining. *ACS Chem. Biol.*, **10**, 2841–2849.
12. Gust,B., Challis,G.L., Fowler,K., Kieser,T. and Chater,K.F. (2003) PCR-targeted *Streptomyces* gene replacement identifies a protein domain needed for biosynthesis of the sesquiterpene soil odor geosmin. *Proc. Natl. Acad. Sci. U.S.A.*, **100**, 1541–1546.

13. Hong,H.-J., Hutchings,M.I., Hill,L.M. and Buttner,M.J. (2005) The role of the novel Fem protein VanK in vancomycin resistance in *Streptomyces coelicolor*. *J. Biol. Chem.*, **280**, 13055–13061.
