# Supplementary figures and images for: Uncovering the unexplored diversity of thioamidated ribosomal peptides in Actinobacteria using the RiPPER genome mining tool
Source: Nucleic Acids Res. 2019 Mar 27;47(9):4624–37. doi: 10.1093/nar/gkz192 (PMC6511847; doi:10.1093/nar/gkz192)

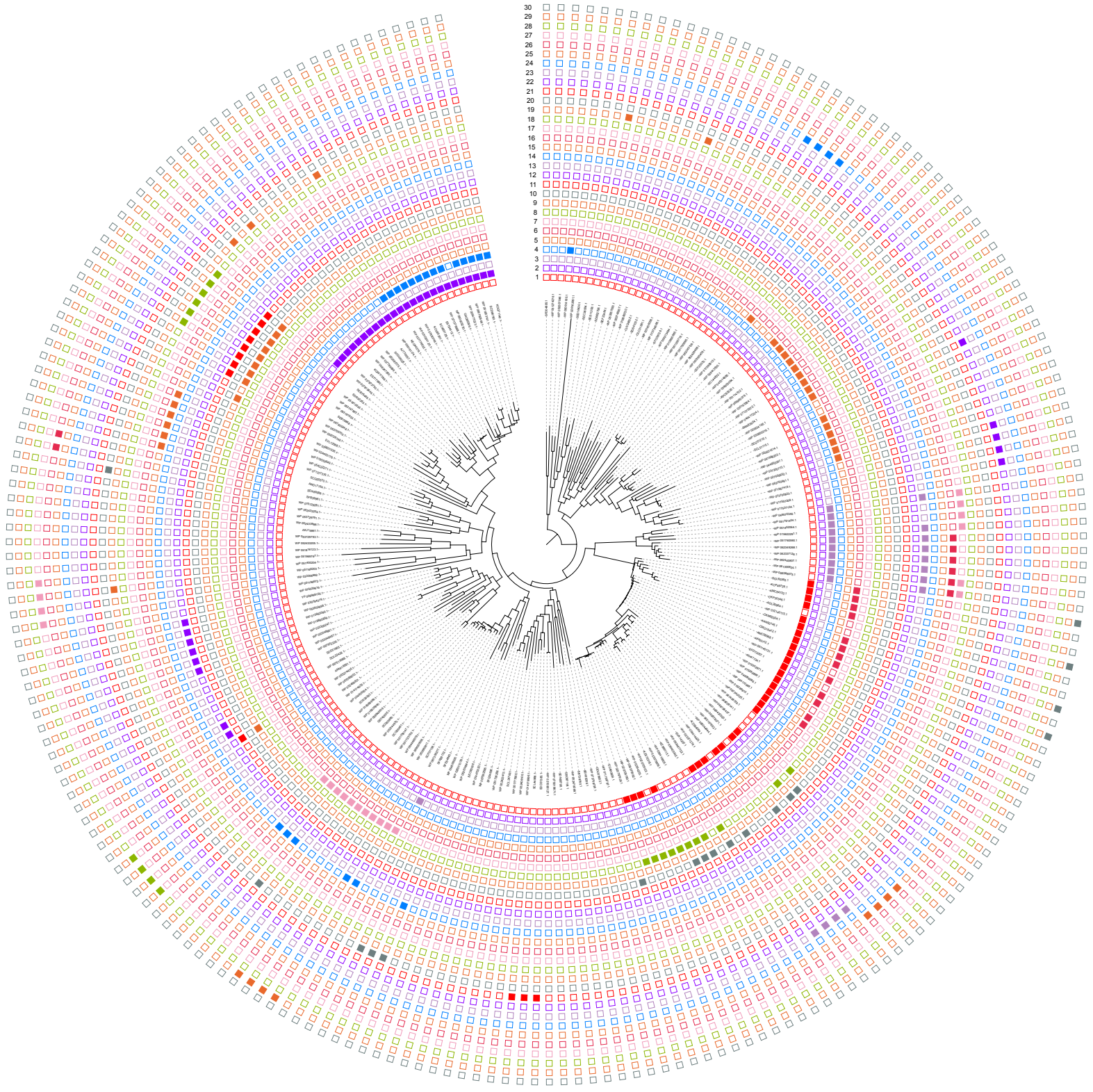

Supplement: Supplementary Data [file gkz192_supplemental_files.zip › Supplementary_Dataset_5.pdf]
